# Supplementary material for: Future hydrogen economies imply environmental trade-offs and a supply-demand mismatch
Source: Nat Commun. 2024 Aug 15;15:7043. doi: 10.1038/s41467-024-51251-7 (PMC11327350; doi:10.1038/s41467-024-51251-7)
Supplement: Supplementary file 1 — Supplementary Information [file 41467_2024_51251_MOESM1_ESM.pdf]

SUPPLEMENTARY INFORMATION FOR:

Future hydrogen economies imply environmental trade-offs and a supply-demand mismatch

Tom Terlouw[1,2,3*], Lorenzo Rosa[4], Christian Bauer[3], and Russell McKenna[2,5*]

Affiliations

[1] Separation Processes Laboratory, Institute of Energy and Process Engineering, ETH Zurich, Zurich 8092, Switzerland

[2] Chair of Energy Systems Analysis, Institute of Energy and Process Engineering, ETH Zurich, Zurich 8092, Switzerland

[3] Technology Assessment Group, Laboratory for Energy Systems Analysis, 5232 Villigen PSI, Switzerland

[4] Department of Global Ecology, Carnegie Institution for Science, Stanford, 94035, CA, United States of America

[5] Laboratory for Energy Systems Analysis, 5232 Villigen PSI, Switzerland

* *Corresponding author: tom.terlouw@psi.ch and russell.mckenna@psi.ch.*

Contents

Supplementary Note 1. System boundaries of non-electrolytic hydrogen production pathways 2

Supplementary Note 2. Optimization problem – hybrid energy systems 4

Supplementary Note 3. Curve-fitting as optimization result for hybrid systems 6

Supplementary Note 4. Review, discussion & limitations 11

Supplementary Note 5. Life cycle inventories 14

Supplementary Note 6. Techno-economic assumptions – data sheet 16

Supplementary Note 7. Environmental burdens from hydrogen production 19

Supplementary Note 8. Additional results 36

SUPPLEMENTARY REFERENCES 43

Supplementary Note 1. System boundaries of non-electrolytic hydrogen production pathways

Supplementary Figure 1 illustrates the system boundaries of our product system with hydrogen production via steam methane reforming (SMR) with (including the red blocks) and without carbon capture and storage (CCS) (excluding the red blocks). All processes are included, from cradle-to-gate up to hydrogen produced with a pressure of 25–30 bar, such as natural gas upgrading, transport, catalyst and adsorbent production, and capturing, transportation, and storage of CO_2 (in case of CCS).

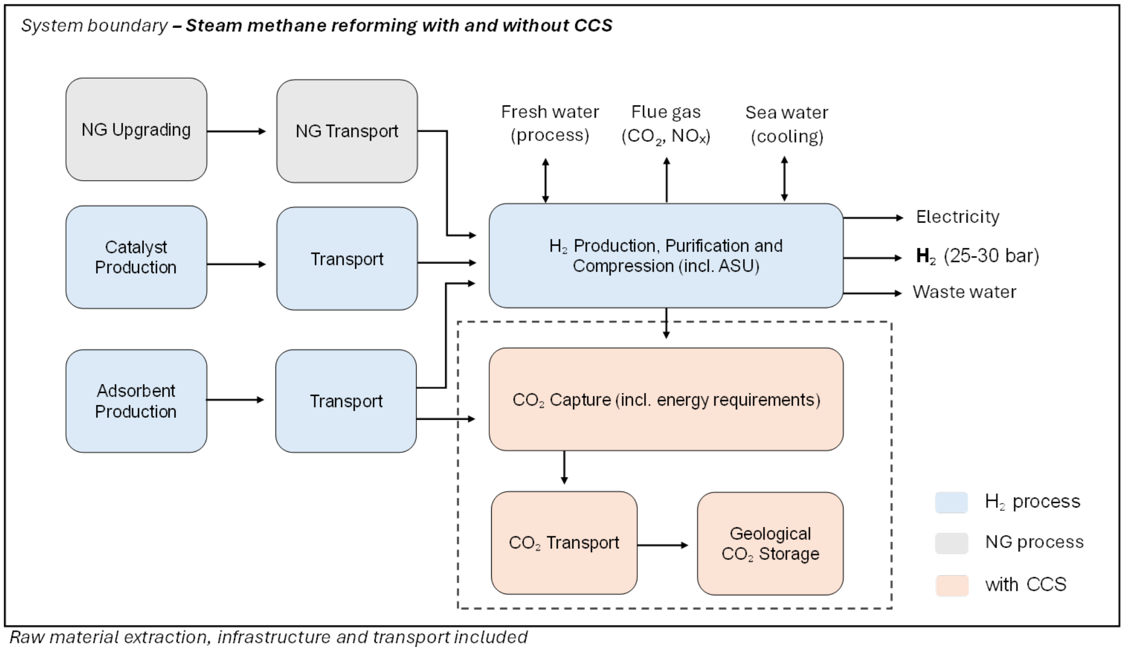

Supplementary Figure 1: System boundaries of hydrogen production *via* steam methane reforming (SMR) with and without carbon capture and storage (CCS), reproduced from Ref[1]. CCS = carbon capture and storage. ASU = air separation unit.

Supplementary Figure 2 shows the system boundaries of our product system with hydrogen production via biomass gasification with (including the red blocks) and without CCS (excluding the red blocks). All processes are included, from cradle-to-gate up to hydrogen produced via biomass gasification with a pressure of 25–30 bar, such as wood sawing, chipping, gasification unit, and capturing, transportation, and storage of CO_2 (in case of CCS).

Supplementary Figure 3 visualizes the system boundaries of our product system with hydrogen production via coal gasification with (including the red blocks) and without CCS (excluding the red blocks). All processes are included, from cradle-to-gate up to hydrogen produced via coal gasification with a pressure of 25–30 bar, such as coal mining, preparation, gasification, and capturing, transportation, and storage of CO_2

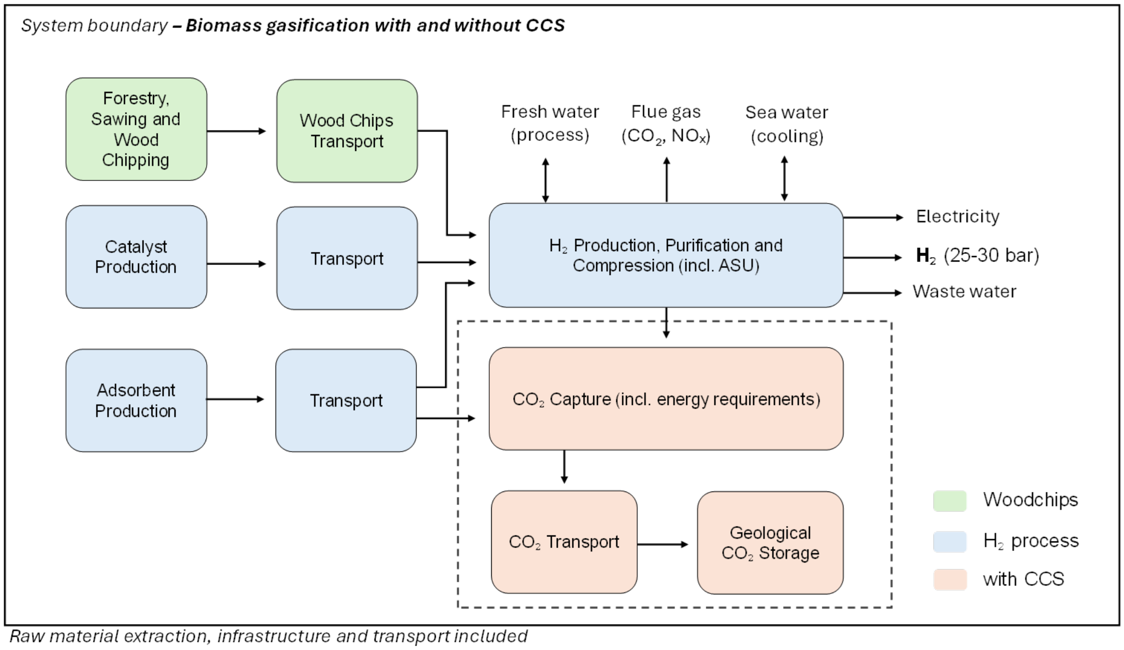

Supplementary Figure 2: System boundaries of hydrogen production *via* biomass gasification with and without carbon capture and storage (CCS), reproduced from Ref[2]. ASU = air separation unit.

(in case of CCS).

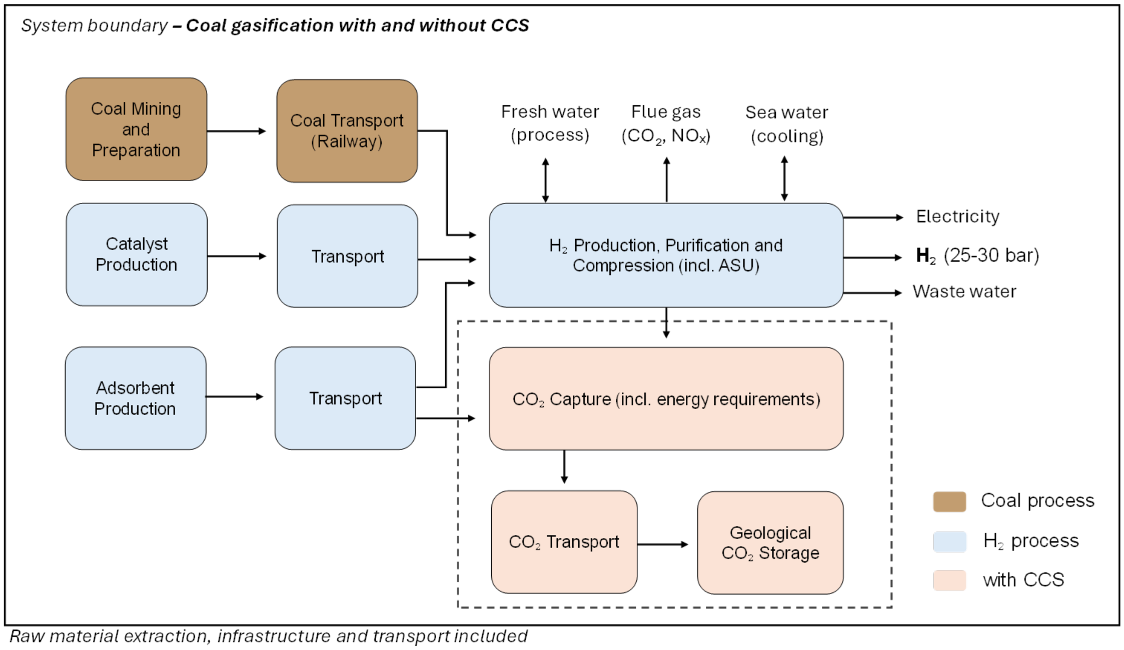

Supplementary Figure 3: System boundaries of hydrogen production *via* coal gasification with and without carbon capture and storage (CCS). ASU = air separation unit.

Supplementary Note 2. Optimization problem – hybrid energy systems

This study considers hybrid energy systems for renewable electricity generation, to be used by the electrolyzer for hydrogen production. To design these hybrid energy systems optimally, a mixed integer linear program is formulated using a similar approach as in Refs.[3–5] The energy system optimization is performed over one year of system operation (T = 8760 hours) for hundred global locations (see Supplementary Figure 7 and Supplementary Note 3. to explain this selection).

The main objective is to design hybrid low-carbon hydrogen production systems with minimal costs, as shown in supplementary equation (1). The total annual cost [€/year] includes the annualized investments (C^{inv}), annualized land costs (C^{land}), annual operation and maintenance costs (C^{om}), where replacements are assumed to be covered in O&M expenditures. It is worth noting that the fuel costs (i.e., electricity) are entirely provided by renewables. Further, land costs are included to account for different space requirements of onshore wind and solar photovoltaic (PV), hence technologies that require more land area are penalized, which is reasonable as these are subsequently used in the geospatial analysis. Land area costs are estimated at around 5,000 euro/ha land (c^{land}, lower end of Refs.[6,7]), on the premise that land can be used for other purposes than renewable energy generation only.

$$\text{minimize} \quad C^{an} = C^{inv} + C^{om} + C^{land}. \tag{1}$$

Supplementary equations (2)–(4) show the underlying cost components as shown in supplementary equation (1).[3,4]

$$C^{inv} = \sum_{i=1}^{M} \frac{\gamma\,(1+\gamma)^{L}}{(1+\gamma)^{L}-1} C_i^{inv}, \tag{2}$$

$$C^{om} = \sum_{i=1}^{M} C_i^{om}, \tag{3}$$

$$C^{land} = \sum_{i=1}^{M} \frac{\gamma\,(1+\gamma)^{L}}{(1+\gamma)^{L}-1} c^{land} \frac{1}{\rho_{\mathrm{i}}} s. \tag{4}$$

Next, we formulate constraints used in the optimization problem.

Energy balance – annual H_2 production

Supplementary equation (5) is introduced to ensure sufficient annual hydrogen production as output from the electrolyzer (here, p_t is the hydrogen energy output from the electrolyzer). The set of time steps is

described as $\mathcal{T} = \{1, 2, \ldots, T\}$.

$$\sum_{t \in \mathcal{T}} p_t \Delta t = 365\mathrm{D}, \tag{5}$$

where D is the daily hydrogen production [kWh/day], Δt denotes the time step, and '365' refers to the annual number of days [day]. The daily hydrogen production is assumed to be 10 tonnes, in line with Ref.[3]

Electrolyzer

Hydrogen (p_t) is produced by polymer electrolyte membrane (PEM) electrolyzers, using renewable electricity (f_t). Here, we assume a constant efficiency, which is in line with earlier findings as shown in supplementary equations (6–7):[8]

$$p_t = \eta f_t, \ \ \forall t, \tag{6}$$

$$0 \leq f_t \leq s, \ \ \forall t. \tag{7}$$

Renewable energy and curtailment

Solar PV, onshore wind, and offshore wind power generation profiles are pre-determined using different Python packages[9–11] (generally denoted by P_t). Their electricity generation (p_t) profile is expressed as in supplementary equation (8):[3,4]

$$p_t \leq \mathrm{P}_t s, \ \ \forall t. \tag{8}$$

The inequality constraint ensures the possibility of curtailing renewable electricity generation.

Battery electricity storage

Battery electricity storage is modeled with a similar approach as Refs[3,4,12]. Please refer to this literature for more explanation. Supplementary equation (9) describes the battery dynamics in terms of energy stored (e_t), considering the battery charging (f_t), discharging (p_t), and a self-discharging factor (Λ).

$$e_t = e_{t-1}(1 - \Lambda \Delta t) + \eta f_t \Delta t - \frac{p_t \Delta t}{\eta}, \ \ \forall t. \tag{9}$$

Supplementary equations (10)–(11) are introduced to prevent simultaneous (dis)charging of the battery (introducing binary variable x_t), and to ensure that the charging and discharging power are within the boundaries of the battery, where a energy charging duration (τ) of 2 hours has been used to size the power capacity of the battery. The terms on the right side of supplementary equations (10) and (11) are bi-linear

and are linearized with similar linear terms as described in Refs[4,13].

Supplementary equation (12) is used to ensure the minimum ($\underline{\delta}$) and maximum state of charge ($\overline{\delta}$) of the battery. Supplementary equation (13) is a periodicity constraint.[13]

$$0 \leq f_t \leq x_t \frac{s}{\tau}, \;\; \forall t, \tag{10}$$

$$0 \leq p_t \leq (1 - x_t) \frac{s}{\tau}, \;\; \forall t, \tag{11}$$

$$\underline{\delta} s \leq e_t \leq \overline{\delta} s, \;\; \forall t, \tag{12}$$

$$e_0 = e_T \tag{13}$$

Next, the optimization is performed for hundred global locations to determine trends with regard to renewable energy capacity and battery capacity installed. These non-linear trend lines are given in the next section.

Supplementary Note 3. Curve-fitting as optimization result for hybrid systems

A curve-fitting approach is used to estimate the optimal design of large-scale hydrogen production facilities. More specifically, non-linear curve fittings are used to quantify the following location-specific aspects: (i) the land share available to install onshore wind and solar PV, (ii) the battery energy storage capacity, and (iii) the electrolyzer capacity. Indeed, different methods exist to address the complexity of optimally designing hydrogen production systems using global scope.

Non-linear curve fittings are a reasonable approach due to their ability to capture non-linear correlations and offer interpretable coefficients, which allow researchers to reuse them. In this way, non-linear curve fittings provide an appropriate balance between computational complexity and the need to determine the (near-)optimal design at each geospatial grid cell (0.25°×0.25°). On the one hand, non-linear curve fittings are typically limited to capturing data outliers, which poses challenges in representing the actual complexity of the system. On the other hand, our analysis does not focus on designing case studies but looks at the global perspective, which makes outliers of individual system designs less important. Clustering might represent an alternative; for example, locations could be clustered based on weather conditions, renewable energy potential, intermittency, and land use. However, determining the optimal number of clusters is complex and likely requires many clusters to capture the set of location-specific aspects globally, potentially leading to an oversimplification of variability within clusters by assuming homogeneity.

The locations for the curve-fitting are specified using a global grid search with 0.5 degree longitudinal

and latitudinal steps. For each wind vs. solar PV electricity ratio (*i.e.,* the values on the x-axis of figures 4–6), twenty case studies were selected across the following segments: 0–1, 1–2, 2–3, 3–4, and 4–5. In this search, only one national case study is (max.) included in each segment (except for ratio 4–5 since there are limited case studies for this segment) to ensure a diverse selection, resulting in twenty different countries per segment. Thus, this results in hundred optimizations per scenario; a total of 400 optimizations for the four scenarios considered. This ratio, as well as the solar PV and onshore wind generation profiles, are calculated using the typical meteorological years function of PVGIS[14]. Python package *pvlib* has been applied to generate PV generation profiles, assuming open-rack ground-mounted multi-Si PV installations[9,10]. In addition, Python package *windpowerlib* is used to calculate wind profiles assuming the power output of a Vestas turbine (V90/2000)[11].

Supplementary Figure 4 shows the correlation between the ratio of annual wind energy and solar energy available (on the x-axis) and the amount of area available for installing onshore wind turbines (on the y-axis) as result of the optimization. The former ratio is determined by dividing the local amount of wind energy available by ground-mounted solar PV energy available. It is worth noting that optimizations are repeated for each future scenario due to different techno-economic assumptions. Supplementary Figure 5 shows the correlation between land share used for electricity generation from onshore wind (on the x-axis) and the energy storage capacity of the battery versus solar PV installed (on the y-axis). Finally, Supplementary Figure 6 visualizes the electrolyzer capacity ratio (on the y-axis) as a function of the land share used for wind electricity production (on the x-axis).

Supplementary Figure 4 and 5 highlight that a non-linear curve fitting has a slightly to significantly better performance than a linear one, thus, we decide to use the non-linear curve fitting to determine land shares available for onshore wind and solar PV. Indeed, the non-linear curve fitting of the battery capacity determination is significantly weaker than the wind share determination. However, we believe that there is a sufficient strong trend that shows higher battery capacity installed with more solar PV capacity installed and with a higher land share for solar PV electricity production, which aligns with findings described in Ref.[4].

For locations with limited data for only one renewable energy technology, the electrolyzer is sized in a way that ensures that all renewable energy peak capacity can be used. This could for example happen at offshore locations or without solar PV or onshore wind data.

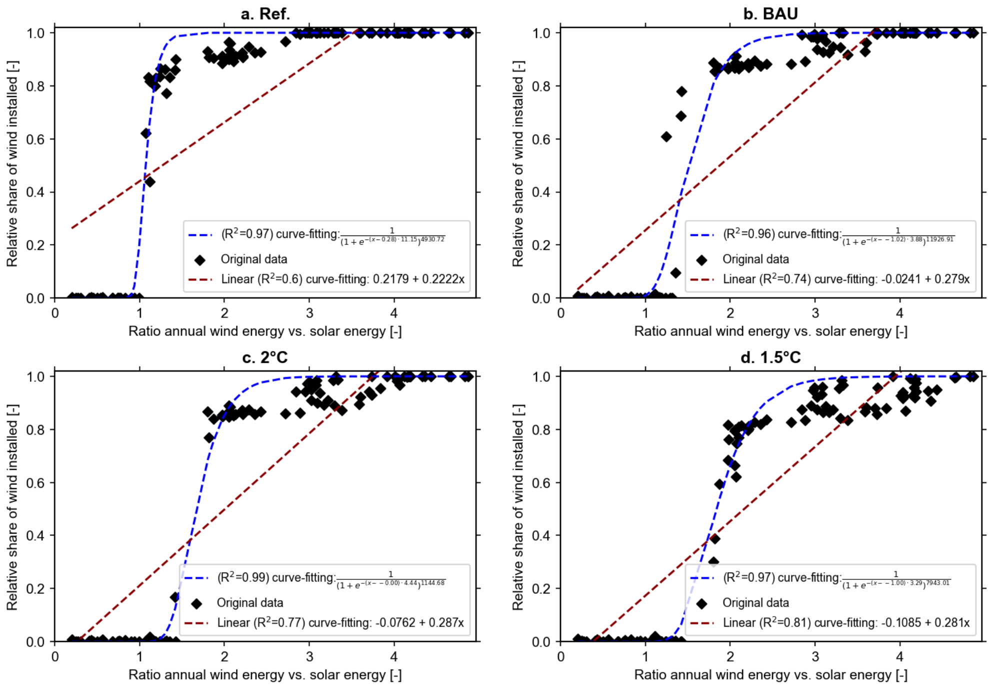

Supplementary Figure 4: Curve-fitting from optimization to determine onshore wind and solar PV land share occupied in a geospatial grid cell for hybrid energy systems, which adds up to '1' (100%) in total, for four scenarios: **a** reference, **b** business-as-usual, **c** $2\,^{\circ}\mathrm{C}$, and **d** $1.5\,^{\circ}\mathrm{C}$. BAU = business-as-usual, Ref. = reference scenario.

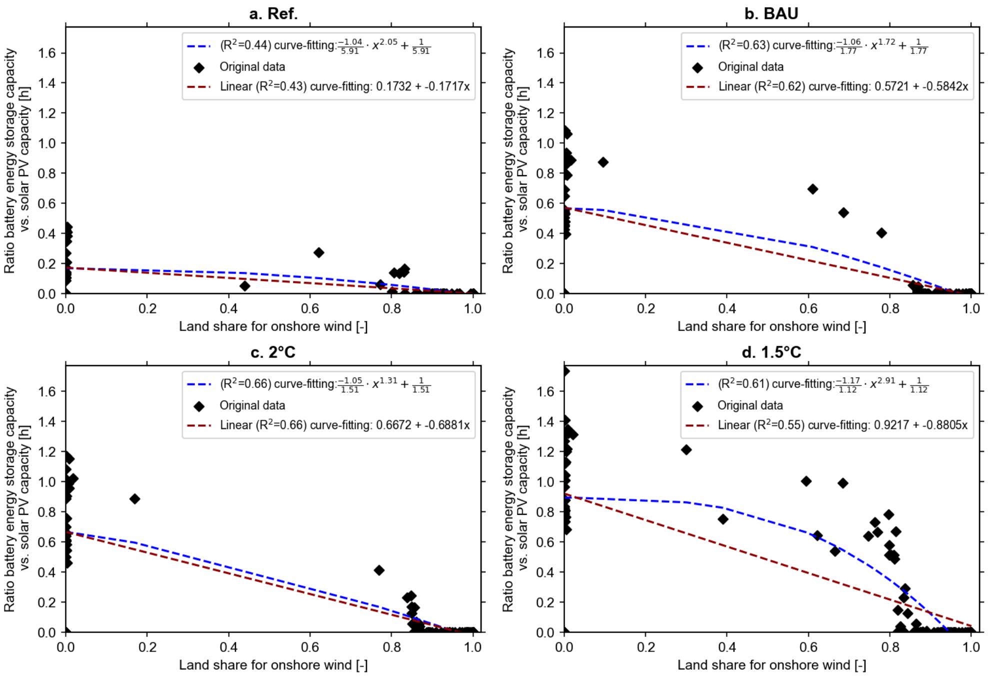

Supplementary Figure 5: Curve-fitting from optimization to determine battery electricity storage capacity installed in hybrid energy systems, for four scenarios: **a** reference, **b** business-as-usual, **c** $2\,°\mathrm{C}$, and **d** $1.5\,°\mathrm{C}$. BAU = business-as-usual, Ref. = reference scenario.

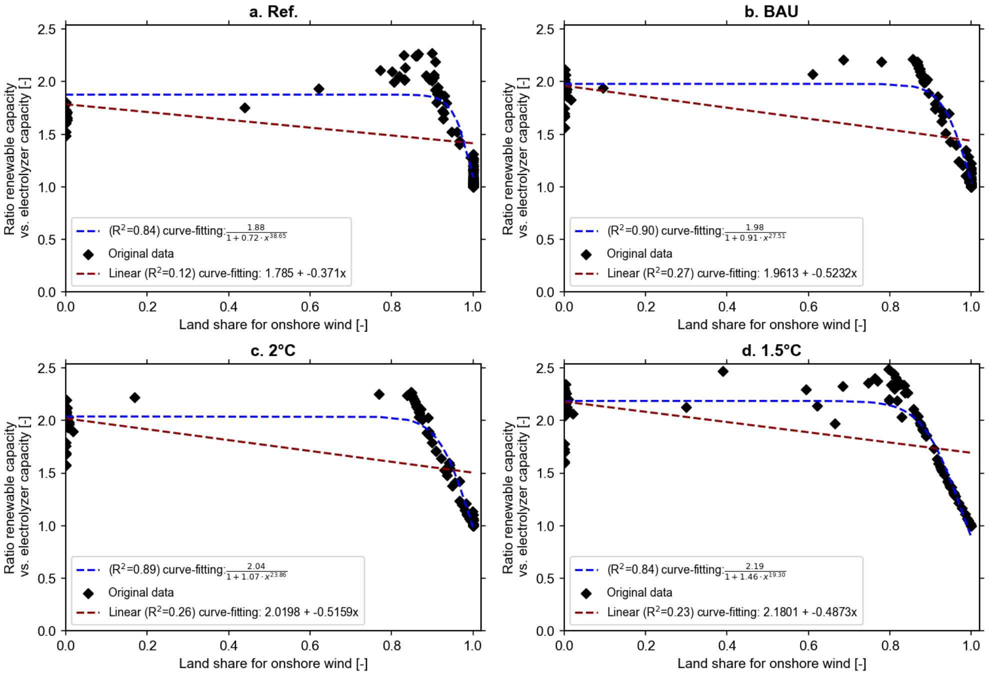

Supplementary Figure 6: Curve-fitting from optimization to determine electrolyzer capacity installed, based on the installed capacity of renewables, in hybrid energy systems, for four scenarios: **a** reference, **b** business-as-usual, **c** $2\,°\mathrm{C}$, and **d** $1.5\,°\mathrm{C}$. BAU = business-as-usual, Ref. = reference scenario.

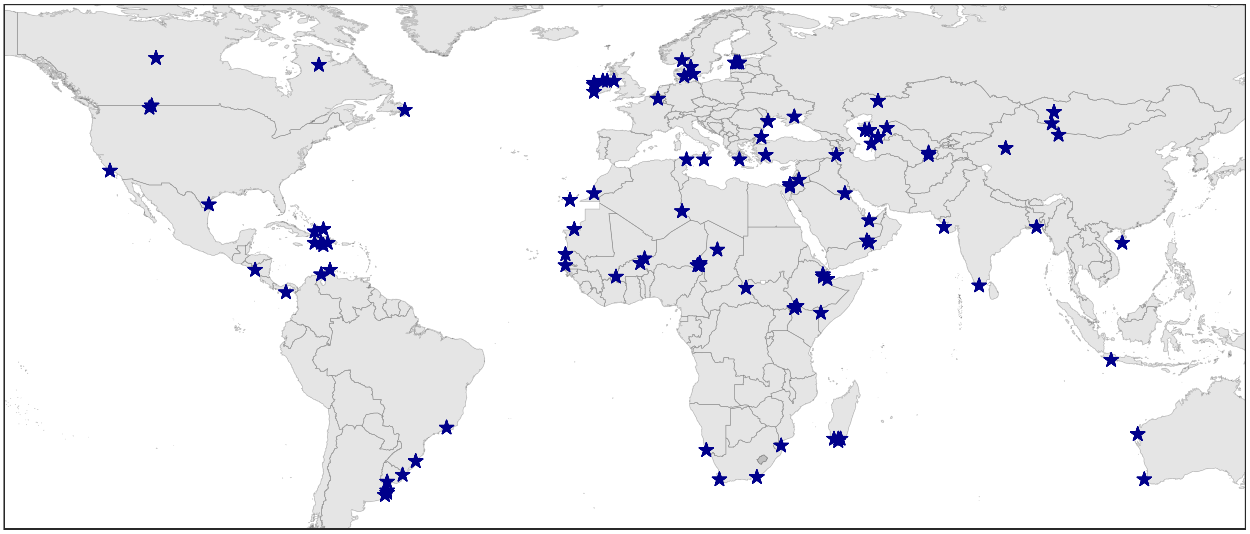

Supplementary Figure 7: Fifty global locations used for the curve-fittings.

Supplementary Note 4. Review, discussion & limitations

In this work, our main novelty is to give indications of the global economic and environmental implications of large-scale hydrogen production in future socio-economic pathways, a short review of current literature on this topic is given below. After that, we discuss the main limitations and suggest potential improvements for future work with a main focus on results and methodological assumptions.

The main body of the article discussed the current limitations of literature on the potential upscaling towards large-scale hydrogen economies. Here, a couple of recent studies on large-scale hydrogen production are discussed. For example, Terlouw et al.[3] provided a comprehensive environmental and techno-economic assessment of optimized hydrogen production now and in the future for five case studies on geographical islands in Europe. These geographical islands exhibit beneficial hydrogen production potential and are used in an analysis upscaling towards 9.5 EJ (per year) hydrogen production *via* water electrolysis using the impacts of these case studies. Hence, they excluded a comprehensive geospatial quantification of location-specific aspects, except for the five case studies, thus, not considering other global regions for hydrogen production potential. A recent study by Weidmer et al.[15] determines the environmental sustainability of future hydrogen economies in 2050 of 500 Mt/hydrogen economies by considering different hydrogen production pathways including grey, blue, and green hydrogen. The impacts are compared to planetary boundaries using prospective life cycle assessment (LCA). While the authors mention that location-specific impacts are crucial, case studies for hydrogen production pathways were used to derive to the impacts of prospective large-scale hydrogen production quotas. Finally, Tonelli et al.[16], incorporated water and land considerations to explore the limitations of a future global hydrogen economy solely focusing on electrolytic hydrogen production using geospatial analysis but considering one socio-economic narrative and excluding the optimal sizing, cost, and other environmental factors.

Thus, prior analyses have been limited by exploring a single socio-economic narrative, while we argue that future hydrogen production scenarios are highly uncertain and depend on socio-economic development pathways. Given the diverse range of possible development scenarios, there is an urgent need for comprehensive assessments that address such uncertainties to determine the potential global environmental implications of future hydrogen production economies. Additionally, there are still environmental burdens and trade-offs that have not been evaluated, such as material utilization and overall trade-offs of hydrogen production pathways. These omissions impede a comprehensive understanding of the potential challenges and opportunities of transitioning to a hydrogen-based economy. Lastly, prior global geospatial analyses have often overlooked the optimal sizing of hybrid hydrogen production systems—considering both regional solar PV and wind potentials—or have done so in a simplified way. Besides, these analyses frequently—in our view unreasonably—assumed the existence of a local power grid in remote regions. Such simplifications typically lead to either under- or over-dimensioning hydrogen production systems and neglecting the con-

sideration of more complex (off-grid) hybrid energy systems with flexibility options, such as batteries and renewable electricity curtailment. We argue that these systems should be designed in a (near-)optimal way using optimization techniques to minimize cost, and to better account for curtailment and battery electricity storage[3,4,12,13].

We quantify a comprehensive set of environmental impact categories, in particular, material utilization and water consumption. Life cycle environmental flows are typically well-defined for greenhouse gas (GHG) emissions. However, some of the material inventories are not well represented in the ecoinvent database. Therefore, we used critical material requirements from literature for onshore wind, offshore wind, and solar PV. For the other technologies, no comprehensive material demand for the critical materials was found, therefore, we aggregated material demands from the ecoinvent database using the specific material flows with 'in ground'. This represents some inconsistency, hence more efforts should be taken to represent better critical material inventories in the ecoinvent database.

As discussed in the main article, some environmental impact categories assessed are very location-specific. In fact, water consumption is calculated per location, however, the environmental water flows throughout supply chains are not tracked. For example, the location-specific production and construction of technologies (such as solar PV) typically exhibit water consumption in other global locations than where the solar PV installation is installed. Indeed, it is challenging to track these water flows, however, future efforts should provide more insights into this issue since we show that water scarcity and consumption are critical aspects of a future low-carbon (hydrogen) economy. One solution is to apply regionalized LCA, which has the potential to keep better track of such global water flows[17,18].

Commodity prices are used in line with the year 2020 (pre-covid). However, the prices of electricity, natural gas, and coal have been subject to significant fluctuations in recent years. Thus, this represents large uncertainties, although the last couple of months show a trend towards pre-covid commodity prices, which supports the prices used for our analysis. Another limitation is the use of a single commodity price for all world regions while commodity prices and inflation are typically very different between world regions, which was not accounted for in our main analysis. However, it is important to highlight that the main analysis is focused on low-carbon hydrogen production, making the prices of fossil fuels less influential.

In this work, no value is given to oxygen as a by-product of water electrolysis as it is typically vented into the atmosphere. In fact, oxygen can be a valuable by-product (*e.g.,* for hospitals) and might have economic value[19], which could make the business case of hydrogen *via* water electrolysis more economically attractive.

The geospatial analysis used pre-defined exogenous assumptions for techno-economic and LCA parameters. The inclusion of endogenous learning into the geospatial analysis could be interesting to consider as hydrogen production could be responsible for a substantial share of global final energy production and therefore the additional installation of key components—such as electrolyzers, wind turbines, and solar PV—have

significant implications for the costs of individual components. However, the actual application of endogenous learning could be challenging due to additional complexities, such as data requirements and model complexity.

We applied a simplified method, in our geospatial analysis, to assess the potential for land utilization and to determine the optimal sizing of energy technologies, such as solar PV and wind capacity. This involved acquiring various land use types and applying corresponding land use factors from various literature sources. Additionally, the ratio of solar PV to wind availability decides on the sizing of the electrolyzer and any required battery systems, which is based on the energy system optimization of a set of hundred case studies for four future scenarios. Optimally designing a hydrogen production site for each individual grid pixel individually would be the most accurate. However, such a method would be computationally intensive, involving calculations for approximately one million grid pixels. Therefore, we used a curve-fitting technique to estimate the land allocation between wind and solar PV, the capacity of the electrolyzer, and the capacity of battery electricity storage. Satisfactory R^2 values are obtained from the curve fits, ranging between 0.6–1. To increase accuracy, we recommend applying energy system optimization when there are fewer case studies. Furthermore, refining these curve fits might be achieved by incorporating a broader set of case studies and data as well as considering different correlations. Importantly, we did not directly account for social aspects in our assessment of potential land use, although some societal considerations are embedded into our land use factors. In fact, social acceptance might represent another significant barrier to the large-scale expansion of renewable electricity.

Single impact factors for other hydrogen production technologies are used to complement hydrogen production mixes based on electrolytic hydrogen production; for biomass-based hydrogen production, coal gasification, and steam methane reforming. However, hydrogen production has location-specific impacts, especially for biomass-based hydrogen production, mainly depending on the biomass source. In our study, the water consumption of 1 kilogram of hydrogen production *via* biomass gasification with carbon capture and storage is around 40–45 kilogram H_2O per kilogram H_2. However, some recent studies report (much) higher water and land footprint of biomass gasification, up to more than 3400 kilogram H_2O per kilogram H_2 in Ref[20]. To illustrate this, applying a higher specific water consumption results in a substantial increase of global water consumption in the $1.5\,^{\circ}\mathrm{C}$ scenario up to 413 bcm for biomass gasification with carbon capture and storage only.

Supplementary Note 5. Life cycle inventories

Supplementary table 1 shows the life cycle inventories used for this study.

Ecoinvent data is used for LCA of wind turbines, ground-mounted solar PV, and residential solar PV (mounted on the roof). Most of the inventories are obtained from the open-source Python package premise (v.2.0.0)[21] with an update for more conservative platinum utilization in PEM electrolyzers considering 0.75 kg/MW[21]. These life cycle inventories are obtained from papers presented in Refs.[1,2,22–24] Water consumption for water electrolysis is assumed to be 24 kg H_2O/kg H_2, considering the stochiometric water requirements (9 kg H_2O/kg H_2 required while 8 kg O_2/kg H_2 is produced), and water losses due to water treatment (15 kg H_2O/kg H_2)[16].

In our geospatial analysis, the autothermal reforming (ATR)+CCS dataset is used to represent natural gas reforming+CCS as the ATR dataset better represents low-carbon blue hydrogen production with higher capture efficiencies of CO_2 than conventional SMR[1]. Further, the ecoinvent database includes an activity for reverse osmosis. However, this activity is updated with recent environmental flow factors found in Ref.[25], which shows that reverse osmosis using seawater has 117–177% higher environmental flows compared to the ecoinvent activity and is considered to be more reliable.

Supplementary Table 1: Life cycle inventories used for activities of hydrogen production. GLO = global. PV = photovoltaic. RER = Europe. RoW = rest of the world. CH = Switzerland. PEM = polymer electrolyte membrane. NMC = nickel manganese cobalt. CCS = carbon capture and storage. MDEA. = methyldiethanolamine.

Technology	Sub/info	Activity	Location	Refs.
**Energy generation**				
Solar PV, ground-mounted		photovoltaic open ground installation, 570 kWp, multi-Si, on open ground	RER	[21,26]
Solar PV, residential		market for photovoltaic slanted-roof installation, 3kWp, multi-Si, panel, mounted, on roof	GLO	[27]
Onshore wind		wind turbine construction, 2MW, onshore<br>& market for network connection, turbine 2MW, onshore	GLO	[27]
Offshore wind		market for wind power plant, 2MW, offshore, fixed parts<br>& market for wind power plant, 2MW, offshore, moving parts	GLO	[27]
**Energy conversion**				
Electrolyzer		electrolyzer production, 1MWe, PEM, Stack & electrolyzer production, 1MWe, PEM, Balance of Plant	RER	[21,24,28]
**Water requirements**				
Deionised water		market for water, deionised	RoW	[27]
Reverse osmosis	Multiplied with factors based on Ref.[25]	tap water production, seawater reverse osmosis, conventional pretreatment, baseline module, single stage, upgraded	GLO	[25,27]
**Hydrogen leakage**				
Biosphere flow		Hydrogen ('air')		[27]
**Battery**				
Battery electricity	energy	Li-ion (NMC) & Battery management system, kWh<br>& Energy management system, kWh	GLO	[3,29,30]
	power	Power conditioning system, container system	GLO	[3,29,30]
**Fossil-fuel based hydrogen**				
Steam methane reforming	premise (v2.0.0)	hydrogen production, steam methane reforming of natural gas, 25 bar	CH	[1,21]
Steam methane reforming with CCS	premise (v2.0.0)	hydrogen production, auto-thermal reforming of natural gas, with CCS (MDEA, 98% eff.), 25 bar	RER	[1,21]
Autothermal reforming	premise (v2.0.0)	hydrogen production, auto-thermal reforming of natural gas, 25 bar	RER	[1,21]
Autothermal reforming with CCS	premise (v2.0.0)	hydrogen production, auto-thermal reforming of natural gas, with CCS (MDEA, 98% eff.), 25 bar	RER	[1,21]
Coal gasification	premise (v2.0.0), water adjusted using Ref.[31]	hydrogen production, gaseous, 30 bar, from hard coal gasification and reforming, at coal gasification plant	RER	[21–23]
Coal gasification with CCS	premise (v2.0.0), water adjusted using Ref.[31]	hydrogen production, gaseous, 30 bar, from hard coal gasification and reforming, with CCS, at coal gasification plant	RER	[21–23]
Wood gasification	premise (v2.0.0)	hydrogen production, gaseous, 25 bar, from heatpipe reformer gasification of woody biomass, at gasification plant	RER	[2,21]
Wood gasification with CCS	premise (v2.0.0)	hydrogen production, gaseous, 25 bar, from heatpipe reformer gasification of woody biomass with CCS, at gasification plant	RER	[2,21]

Supplementary Note 6. Techno-economic assumptions – data sheet

Generic commodity prices are used for natural gas, coal, and biomass, which are 23 euro/MWh, 13 euro/MWh, and 5.4 euro/MWh, respectively[32–34]. The 1.5°C scenario of IRENA applies the same techno-economic data as 1.5°C of REMIND. Cost data refers to literature data and estimations by the authors. Further, additional cost reductions and improvements are expected for a more ambitious climate scenario due to expected technology learning of low-carbon technologies towards a decarbonized energy system. min. = minimum. max. = maximum.

Supplementary Table 2: Techno-economic parameters used in calculating hydrogen production costs and impacts. These techno-economic parameters are selected from a wide body of literature sources. The cost figures refer to the reference and future scenarios for 2050. CCS = carbon capture and storage. PV = photovoltaic. SoC = state of charge. O&M = operation & maintenance. CAPEX = capital expenditures. Eff. = efficiency. Disch. = discharge.

Technology sheet	sub	Reference (2022)	Baseline (2050)	2°C (2050)	1.5°C (2050)	Unit	Refs.
**Solar PV – residential**	Power density	167	225	250	275	MW/km^2	[35]
	CAPEX	1800	1000	750	550	euro/kW	[30]
	O&M	0.01	0.01	0.01	0.01	-	[30]
	Lifetime	30	32.5	35	37.5	years	[30]
**Solar PV – ground-mounted**	Power density	73	83	92	101	MW/km^2	[35,36]
	CAPEX	1000	600	450	300	euro/kW	[3,30,37]
	O&M	0.01	0.01	0.01	0.01	-	[3,30,37]
	Lifetime	30	32.5	35	37.5	years	[3,30]
**Onshore wind**	Power density	9	11	13	15	MW/km^2	[35,38]
	CAPEX	1500	1200	1050	900	euro/kW	[3,30,37,39]
	O&M	0.03	0.03	0.03	0.03	-	[3,30,37,39]
	Lifetime	20	22.5	25	27.5	years	[3,30]
**Offshore wind**	Power density	6	5	6	7	MW/km^2	[35]
	CAPEX	3000	2000	1750	1500	euro/kW	[3,30,37,39]
	O&M	0.03	0.03	0.03	0.03	-	[3,30,37,39]
	Lifetime	20	22.5	25	27.5	years	[3,30]
**Electrolyzer**	CAPEX	1250	900	700	500	euro/kW	[3,30,40]
	Eff.	0.6	0.65	0.675	0.7	-	[3,30,40]
	Lifetime	7	8	9	10	years	[3,30]
	O&M	0.05	0.05	0.05	0.05	-	[3,30]
**Battery**	CAPEX	275	160	120	80	euro/kWh	[3,30]
	O&M	0.02	0.02	0.02	0.02	-	[3,30]
	Lifetime	13	13	14	15	years	[3,30]
	Charge rate	2	2	2	2	h	[30]
	Depth of disch.	0.93	0.93	0.93	0.93	-	[3,12]
	SoC (min.)	0.035	0.035	0.035	0.035	-	[3,12]
	SoC (max.)	0.965	0.965	0.965	0.965	-	[3,12]
	Discharge eff.	0.954	0.954	0.954	0.954	-	[3,12]
	Charging eff.	0.954	0.954	0.954	0.954	-	[3,12]
	Self-discharging losses	0.00054	0.00054	0.00054	0.00054	-	[3,12]
**Coal gasification without CCS**	CAPEX	2670	2670	2670	2670	euro/kW	[41]
	Eff.	0.6	0.6	0.6	0.6	-	[41]
	O&M	0.05	0.05	0.05	0.05	-	[41]
	Lifetime	25	25	25	25	years	[41]
**Coal gasification with CCS**	CAPEX	2780	2780	2780	2780	euro/kW	[41]
	Eff.	0.58	0.58	0.58	0.58	-	[41]
	O&M	0.05	0.05	0.05	0.05	-	[41]
	Lifetime	25	25	25	25	years	[41]
**SMR without CCS**	CAPEX	910	910	910	910	euro/kW	[41]
	Eff.	0.76	0.76	0.76	0.76	-	[41]
	O&M	0.047	0.047	0.047	0.047	-	[41]
	Lifetime	25	25	25	25	years	[41]
**SMR wit CCS**	CAPEX	1680	1360	1360	1280	euro/kW	[41]
	Eff.	0.69	0.69	0.69	0.69	-	[41]
	O&M	0.03	0.03	0.03	0.03	-	[41]
	Lifetime	25	25	25	25	years	[41]
**Biomass gasification without CCS**	CAPEX	3000	2500	2200	1900	euro/kW	[42]
	Eff.	0.61	0.61	0.61	0.61	-	[42]
	O&M	0.1	0.1	0.1	0.1	-	[42]
	Lifetime	25	25	25	25	years	[42]
**Biomass gasification with CCS**	CAPEX	4300	3800	2700	2300	euro/kW	[42]
	Eff.	0.55	0.55	0.55	0.55	-	[42]
	O&M	0.1	0.1	0.1	0.1	-	[42]
	Lifetime	25	25	25	25	years	[42]
**H_2 leakage**	-	0.025	0.025	0.025	0.025	-	Lower end Ref.[16]

Supplementary Table 3: Suitability factors used for different land use types. na/NaN = not available/applicable. PV = photovoltaic. Refs. = references.

	solar PV	Wind	Refs.
**forest**	0	0.1	Estimated based on Refs.[16,43]
**agri**	0.1	0.5	Estimated based on Refs.[16,43]
**urban**	0.1	0	Estimated based on Refs.[16,43]
**open**	0.1	0.8	Estimated based on Refs.[16,43]
**low vegetation**	0.1	0.7	Estimated based on Refs.[16,43]
**na**	NaN	NaN	
**sea**	0	0.7	Estimated based on Refs.[44]

Supplementary Note 7. Environmental burdens from hydrogen production

This section provides a contribution analysis for (potentially) low-carbon hydrogen production pathways.

Supplementary Figure 8 shows the climate change impacts for the reference year 2022 on the y-axis of four different electrolytic hydrogen production configurations and two highly common hydrogen production pathways based on natural gas on the x-axis: ATR and SMR with and without CCS. The size and color of the bar segments correspond to the different processes required for the supply chain of hydrogen production from cradle-to-gate. The total climate change impacts are reported above the bar segments.

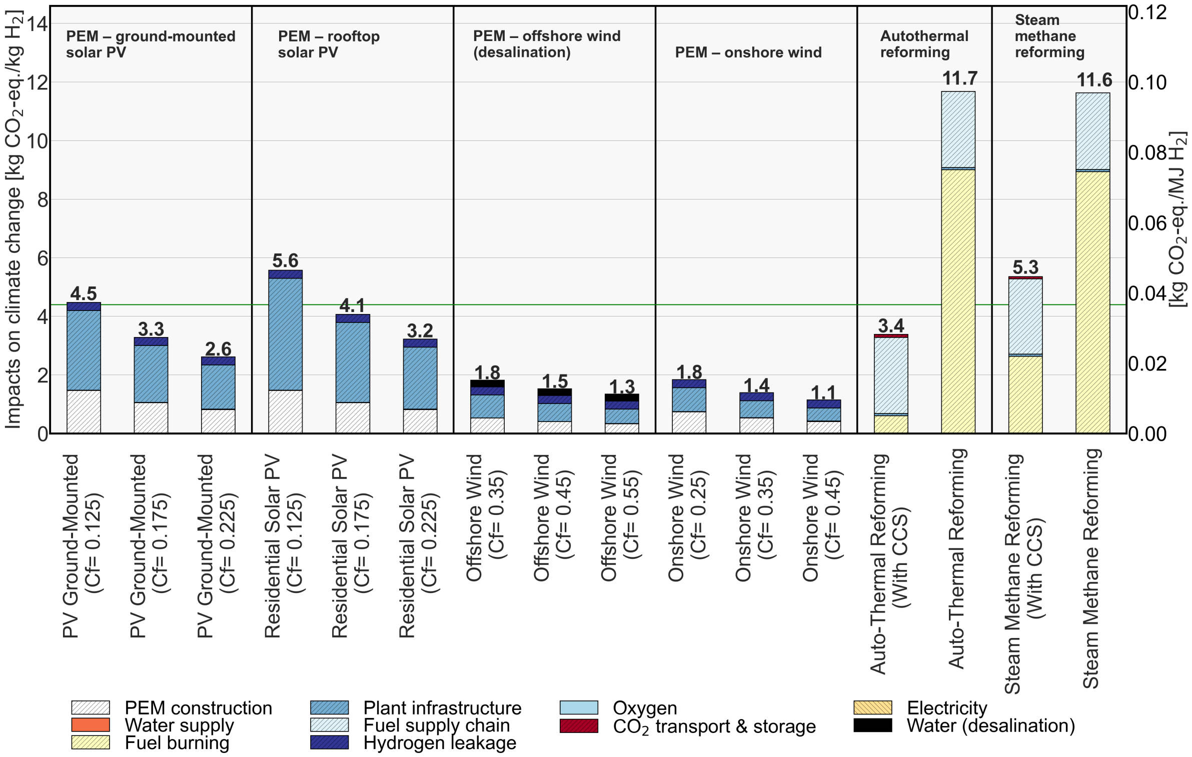

Supplementary Figure 8: Life cycle GHG emissions from hydrogen production pathways using the reference scenario in 2022. The green horizontal line shows the green hydrogen production level set by CertifHy (4.4 kg CO_2-eq. kg^{-1} H_2)[45]. Cf = capacity factor. CCS = carbon capture and storage. PV = photovoltaic. Here, energy storage is excluded from these life cycle inventories but is (using batteries) included in our global analysis for hybrid energy systems. The negative emissions (no carbon removal, but avoidance) from autothermal and steam methane reforming are the result of applying a substitution credit for the generation of electricity, which has been produced as a by-product of these hydrogen production processes.

The capacity factor of renewables is decisive for low-carbon hydrogen production. With low capacity factors of renewables, climate change impacts can cross the ‘green‘ hydrogen production level set by CertifHy (4.4 kg CO_2-eq. kg^{-1} H_2)[45]. Electrolytic hydrogen production pathways generally exhibit much lower GHG

emissions (50–90%) compared to ATR and SMR. These results imply that using a global single life cycle emission factor—*e.g.,* per unit of energy—for renewables is inappropriate to capture global environmental burdens and, thus, unsuitable for geospatial analyses applying a global scope. Applying low-carbon future energy scenarios (in the foreground and background) results in much lower GHG emissions of less than 1.4 kg CO_2-eq. kg^{-1} H_2 for renewable-based hydrogen production routes. Thus, changing the background of the global economy has a substantial influence on individual impacts of hydrogen production[21].

In addition, several common fossil-fuel-based hydrogen production pathways (SMR and ATR) exhibit substantial climate change impacts (more than 11 kg CO_2-eq. kg^{-1} H_2) due to the use of natural gas, which can be reduced by capturing CO_2 from the hydrogen production process; also known as blue hydrogen. Overall, Supplementary Figure 9 illustrates that there is no hydrogen production route that comes without environmental trade-offs, although hydrogen production with highly abundant wind energy sources seems to be the best option from an environmental perspective. This implies that the assessment of hydrogen production pathways should assess life cycle impacts, hence not only focusing on the operational phase.

Supplementary Figure 9 visualizes the environmental burdens of the latter hydrogen production configurations with electrolytic hydrogen production routes on the first row and fossil-fuel-based hydrogen production routes on the second row. The environmental burdens are normalized to the highest environmental burdens per impact category for the eight configurations considered. The latter figure highlights environmental trade-offs between selected hydrogen production routes. Electrolytic hydrogen production pathways generally exhibit much lower GHG emissions compared to ATR and SMR. However, solar-based hydrogen production exhibits substantial environmental trade-offs regarding human toxicity, eutrophication, water, land, materials and minerals, and acidification, mainly due to the production of solar PV panels. These environmental trade-offs can be largely avoided with wind-based hydrogen production, although this might exhibit substantial burdens on ozone depletion and human toxicity due to water desalination and the plant infrastructure, respectively. ATR and SMR also show some trade-offs, especially on non-renewable energy sources, photochemical oxidant formation, ozone depletion, and climate change; the latter can be reduced with CCS. Thus, there is no hydrogen production route that comes without environmental trade-offs, although hydrogen production with highly abundant wind energy sources seems to be the best option from an overall environmental perspective.

Contribution analysis future scenarios – climate change

Supplementary Figure 10 and 11 are contribution analyses on climate change for two future development scenarios with each bar segment representing an activity of the supply chain for hydrogen production.

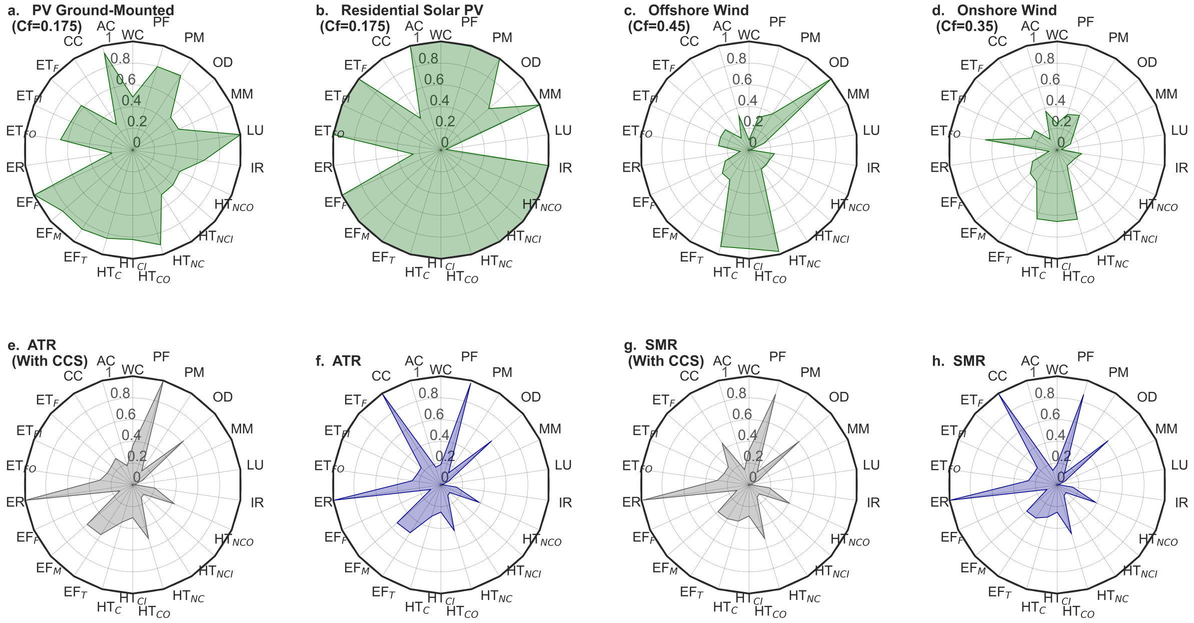

Supplementary Figure 9: Complete set of life cycle environmental burdens from green hydrogen production (first row, green colors) routes compared fossil-fuel-based routes with (blue hydrogen, blue colors) and without CCS (second row, grey colors). Hydrogen production via: **a**. PEM water electrolysis using ground-mounted solar PV electricity. **b**. PEM water electrolysis using residential solar PV electricity. **c**. PEM water electrolysis using offshore wind electricity. **d**. PEM water electrolysis using onshore wind electricity. **e**. Autothermal reforming with CCS. **f**. Autothermal reforming. **g**. Steam methane reforming with CCS. **h**. Steam methane reforming. LU = land use (quality). AC = acidification. CC = climate change. ET_F = ecotoxicity: freshwater. ET_{FI} = ecotoxicity: freshwater, inorganics. ET_{FO} = ecotoxicity: freshwater, organics. ER = energy resources: non-renewable. EF_F = eutrophication: freshwater. EF_M = eutrophication: marine. EF_T = eutrophication: terrestrial. HT_C = human toxicity: carcinogenic. HT_{CI} = human toxicity: carcinogenic, inorganics. HT_{CO} = human toxicity: carcinogenic, organics. HT_{NC} = human toxicity: non-carcinogenic. HT_{NCO} = human toxicity: non-carcinogenic, organics. HT_{NCI} = human toxicity: non-carcinogenic, inorganics. IR = ionising radiation: human health. MM = material resources: metals/minerals. OD = ozone depletion. PM = particulate matter formation. PF = photochemical oxidant formation: human health. WU = water use. Cf = capacity factor. CCS = carbon capture and storage. SMR = steam methane reforming. ATR = autothermal reforming. PV = photovoltaic.

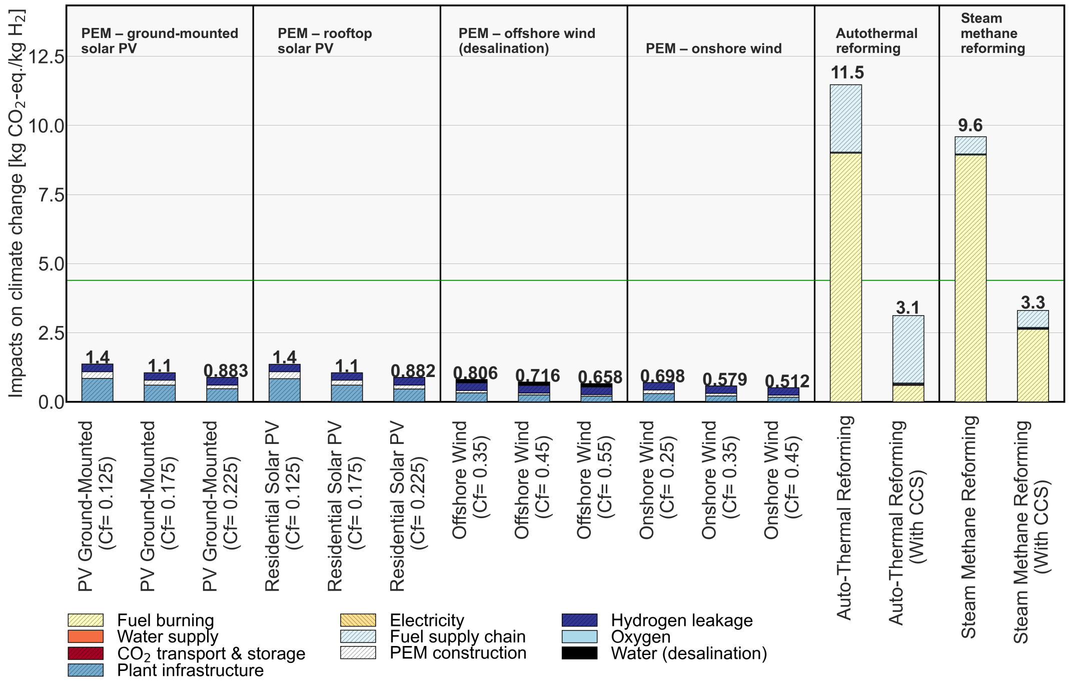

Supplementary Figure 10: Contribution analysis on climate change impact for the $2\,^{\circ}\mathrm{C}$ scenario. Cf = capacity factor. CCS = carbon capture and storage. PEM = polymer electrolyte membrane. PV = photovoltaic.

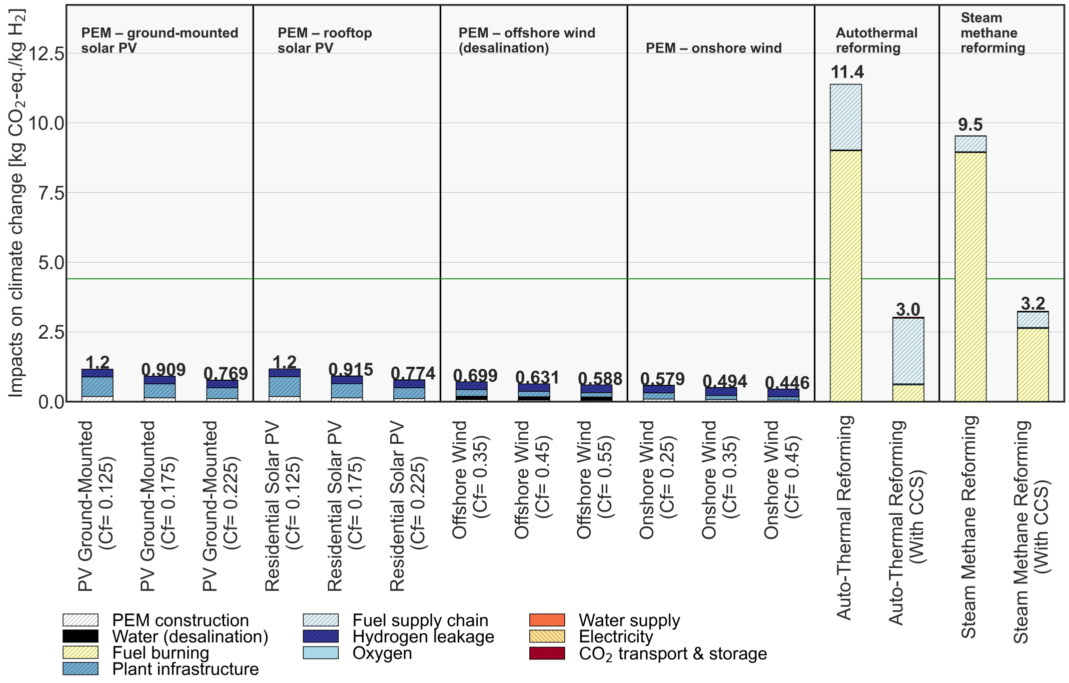

Supplementary Figure 11: Contribution analysis on climate change impact for the $1.5\,^{\circ}\mathrm{C}$ scenario. Cf = capacity factor. CCS = carbon capture and storage. PEM = polymer electrolyte membrane. PV = photovoltaic.

Contribution analysis reference scenario

Contribution analyses of all impact categories are given for the reference scenario in Supplementary Figures 12–32.

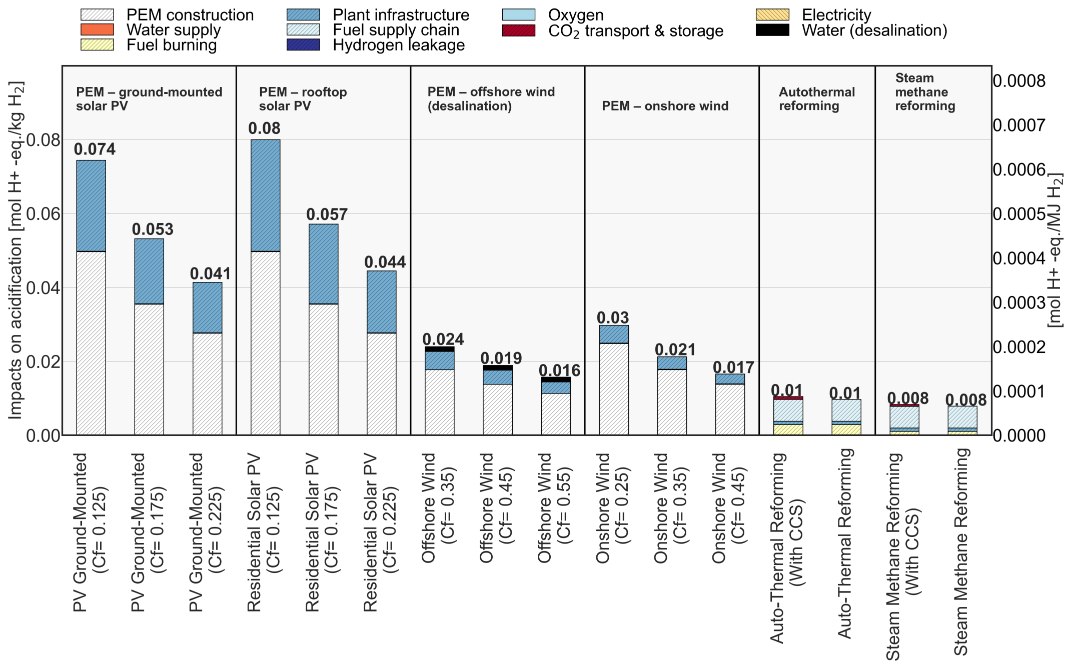

Supplementary Figure 12: Contribution analysis on acidification. Cf = capacity factor. CCS = carbon capture and storage. PEM = polymer electrolyte membrane. PV = photovoltaic.

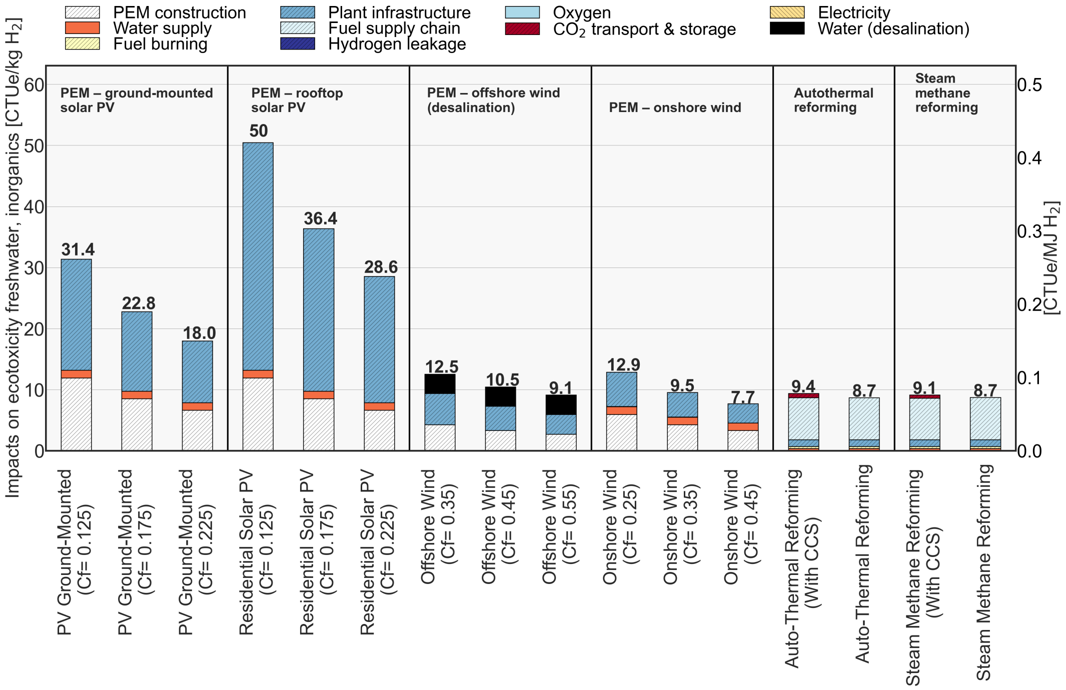

Supplementary Figure 13: Contribution analysis on ecotoxicity, freshwater inorganics. Cf = capacity factor. CCS = carbon capture and storage. PEM = polymer electrolyte membrane. PV = photovoltaic.

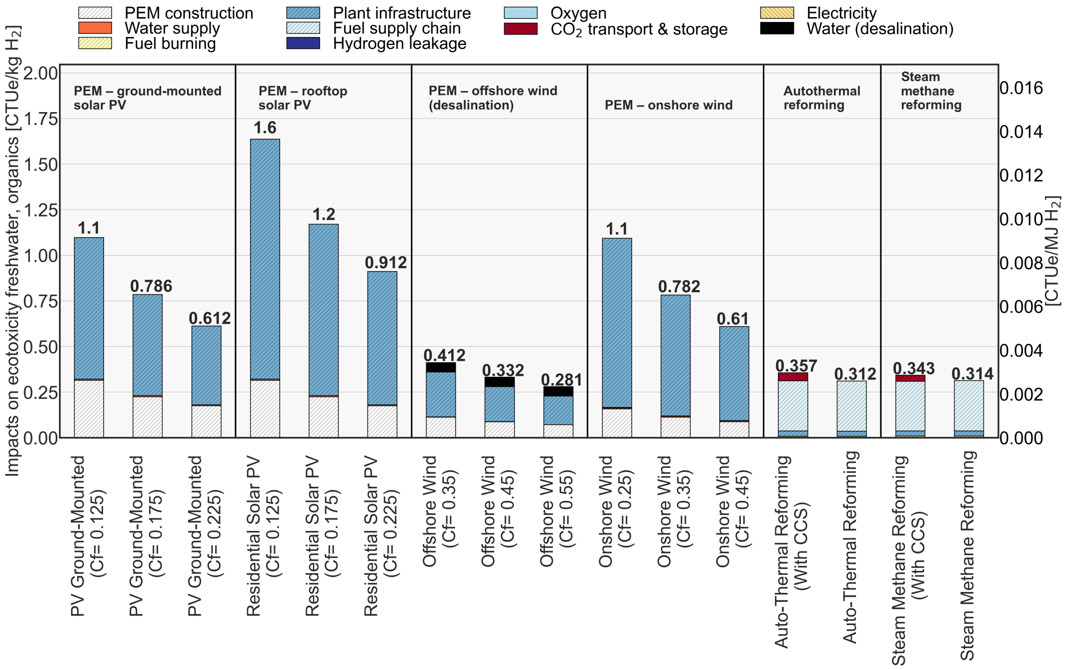

Supplementary Figure 14: Contribution analysis on ecotoxicity, freshwater organics. Cf = capacity factor. CCS = carbon capture and storage. PEM = polymer electrolyte membrane. PV = photovoltaic.

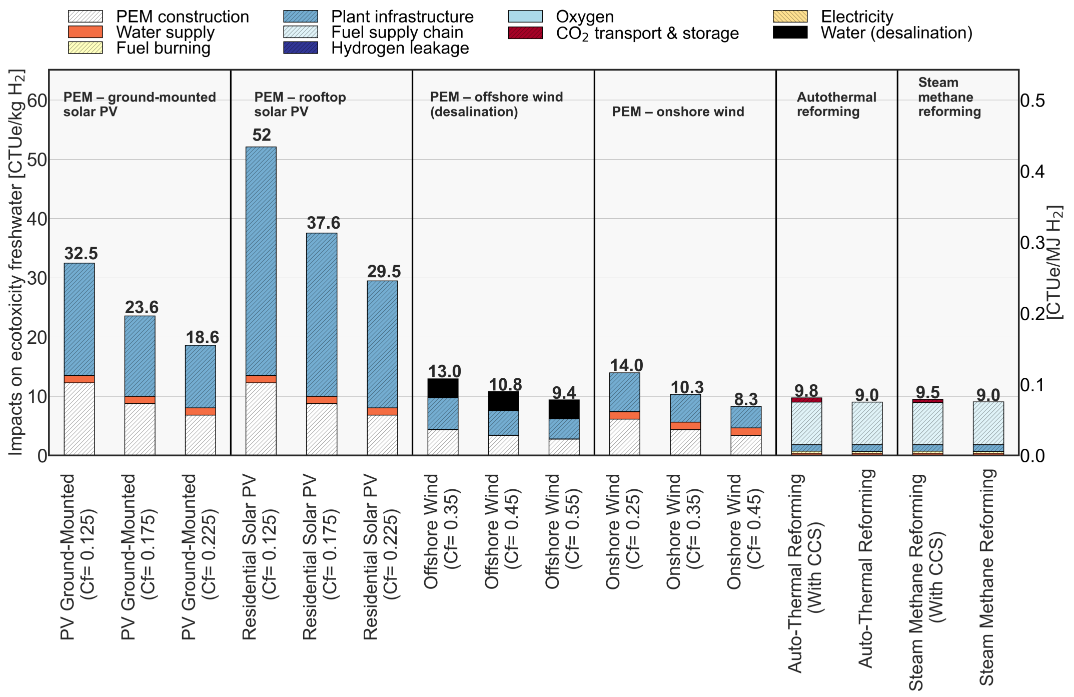

Supplementary Figure 15: Contribution analysis on ecotoxicity, freshwater. Cf = capacity factor. CCS = carbon capture and storage. PEM = polymer electrolyte membrane. PV = photovoltaic.

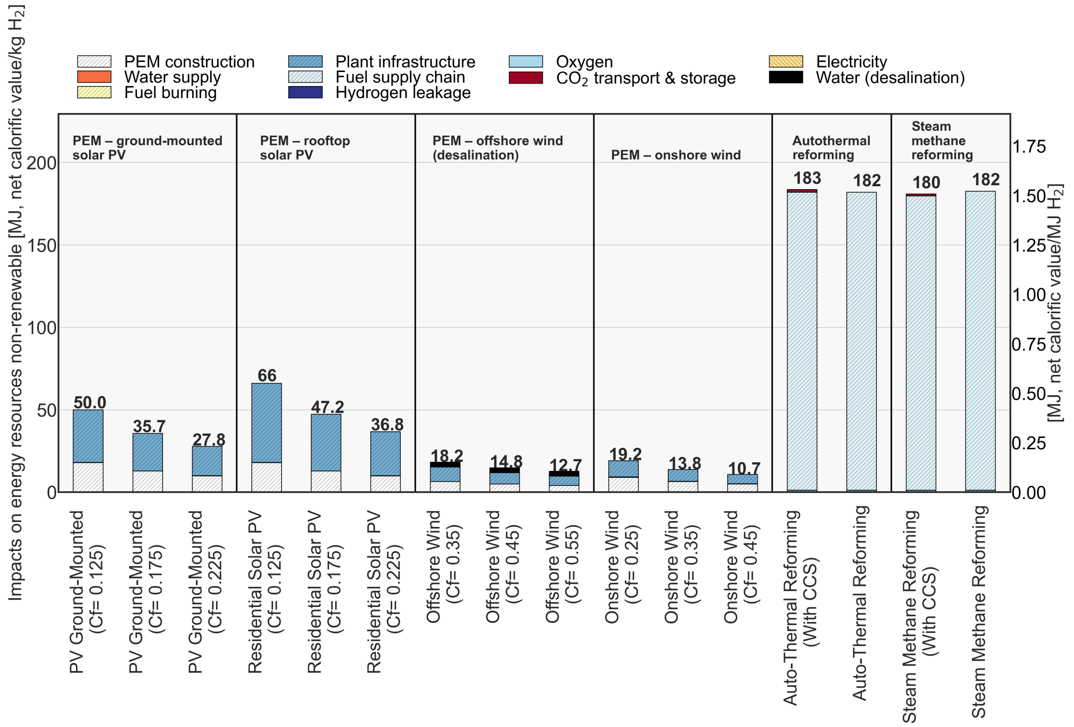

Supplementary Figure 16: Contribution analysis on energy sources, non-renewable. Cf = capacity factor. CCS = carbon capture and storage. PEM = polymer electrolyte membrane. PV = photovoltaic.

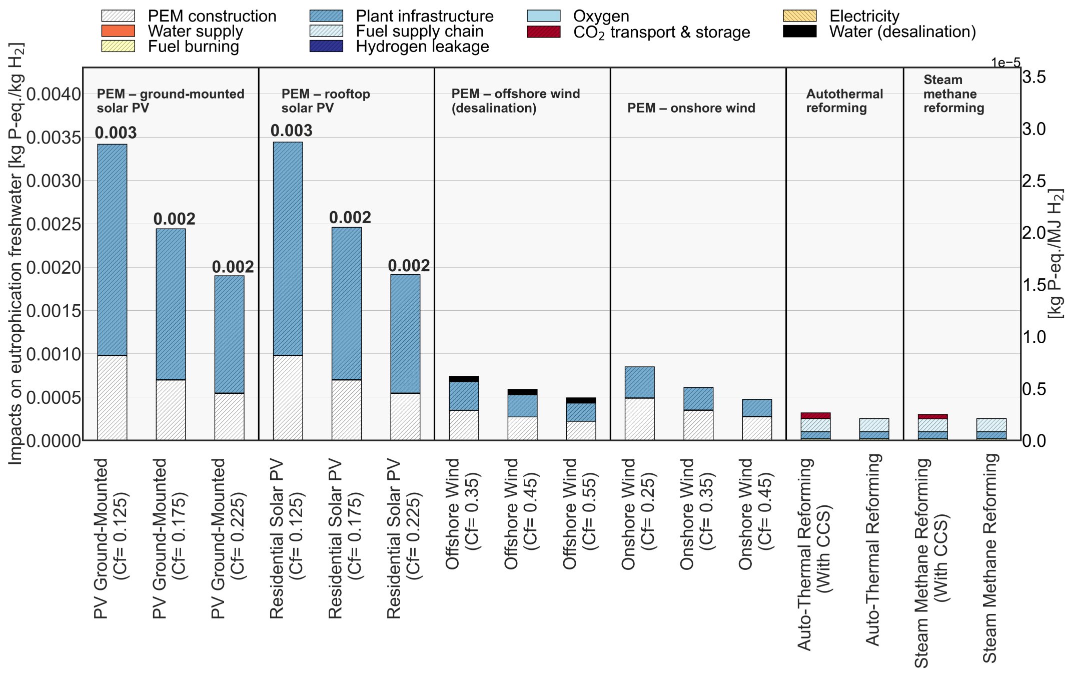

Supplementary Figure 17: Contribution analysis on eutrophication, freshwater. Cf = capacity factor. CCS = carbon capture and storage. PEM = polymer electrolyte membrane. PV = photovoltaic.

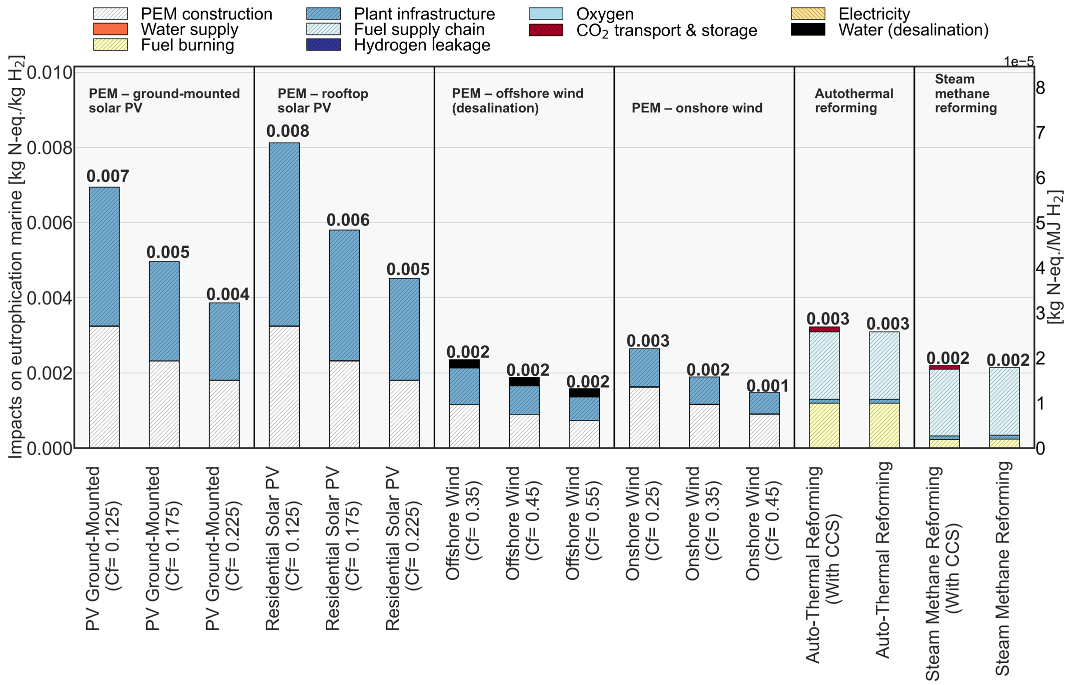

Supplementary Figure 18: Contribution analysis on eutrophication, marine. Cf = capacity factor. CCS = carbon capture and storage. PEM = polymer electrolyte membrane. PV = photovoltaic.

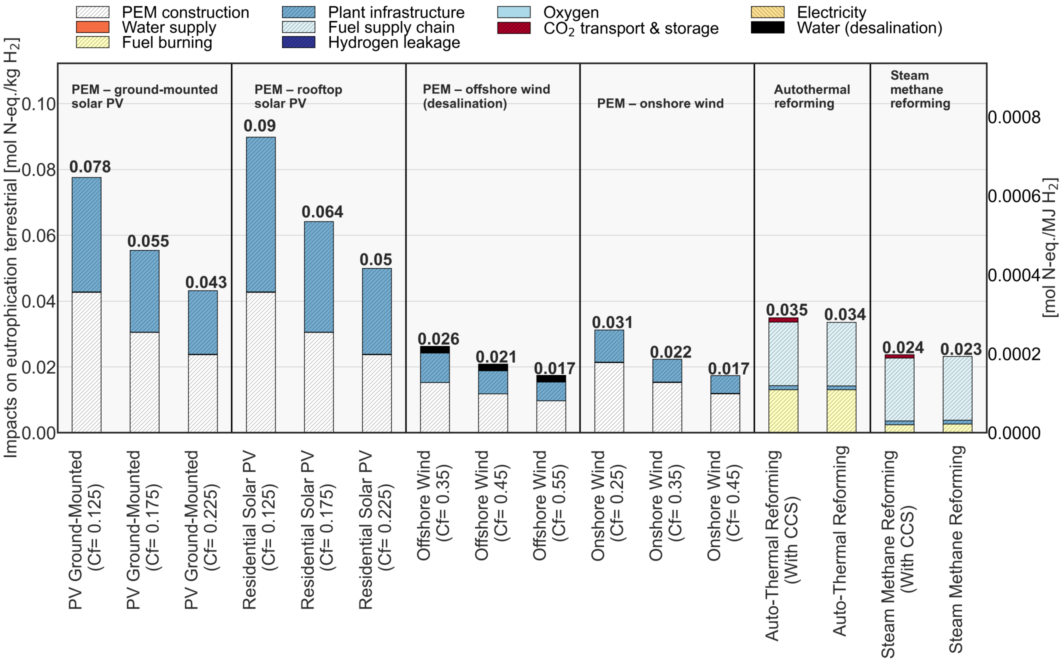

Supplementary Figure 19: Contribution analysis on eutrophication, terrestrial. Cf = capacity factor. CCS = carbon capture and storage. PEM = polymer electrolyte membrane. PV = photovoltaic.

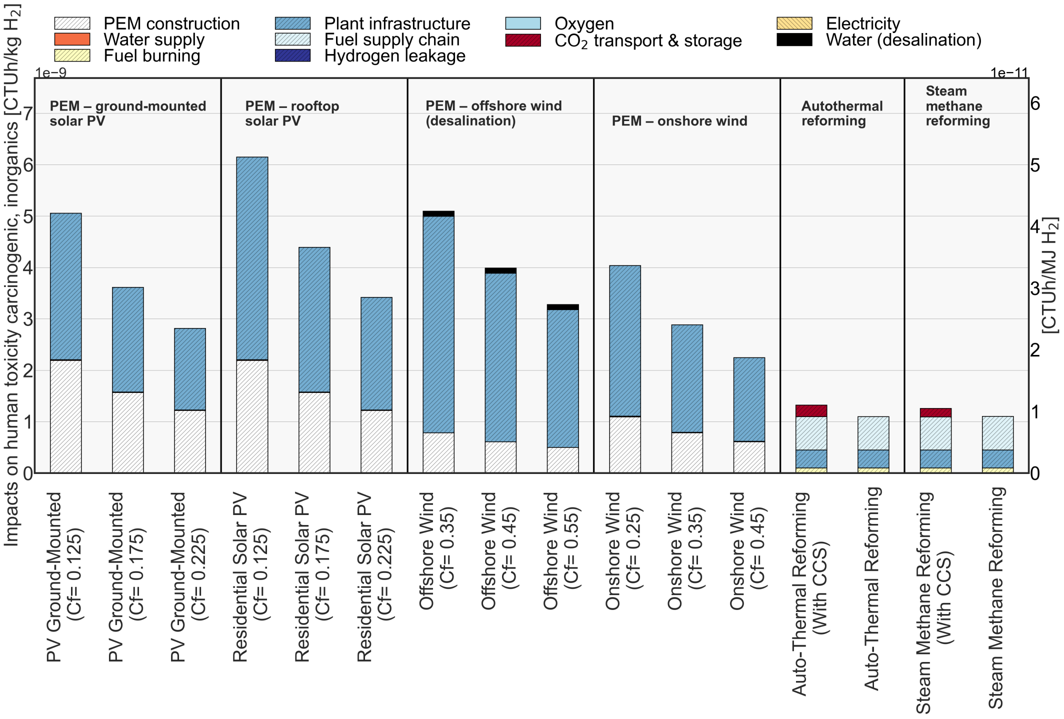

Supplementary Figure 20: Contribution analysis on human toxicity, carcinogenic, inorganics. Cf = capacity factor. CCS = carbon capture and storage. PEM = polymer electrolyte membrane. PV = photovoltaic.

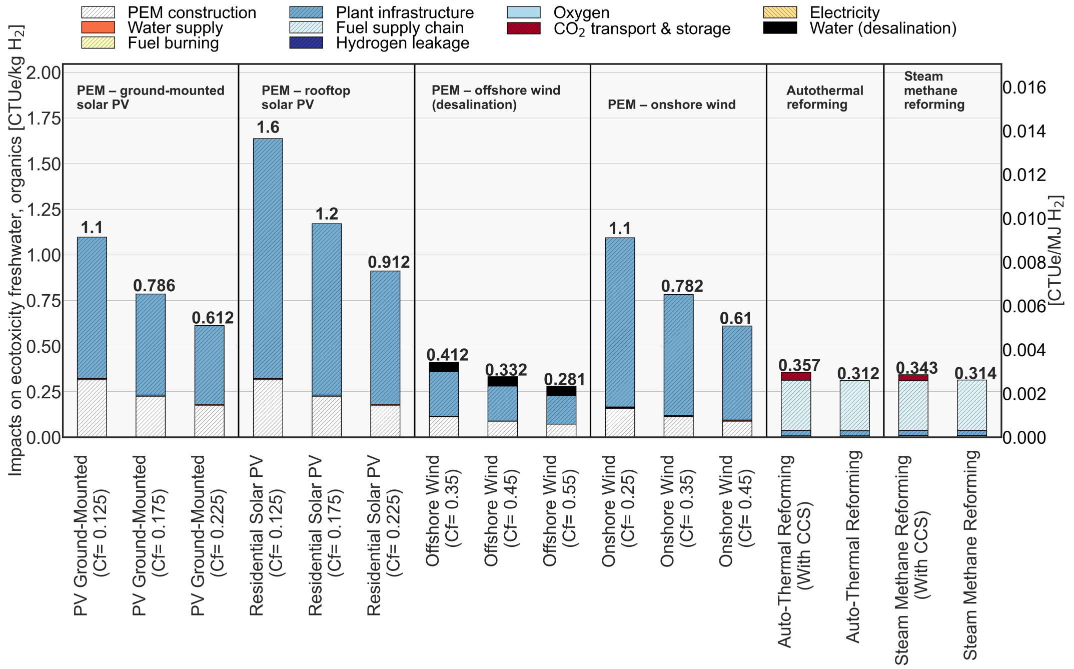

Supplementary Figure 21: Contribution analysis on human toxicity, carcinogenic, organics. Cf = capacity factor. CCS = carbon capture and storage. PEM = polymer electrolyte membrane. PV = photovoltaic.

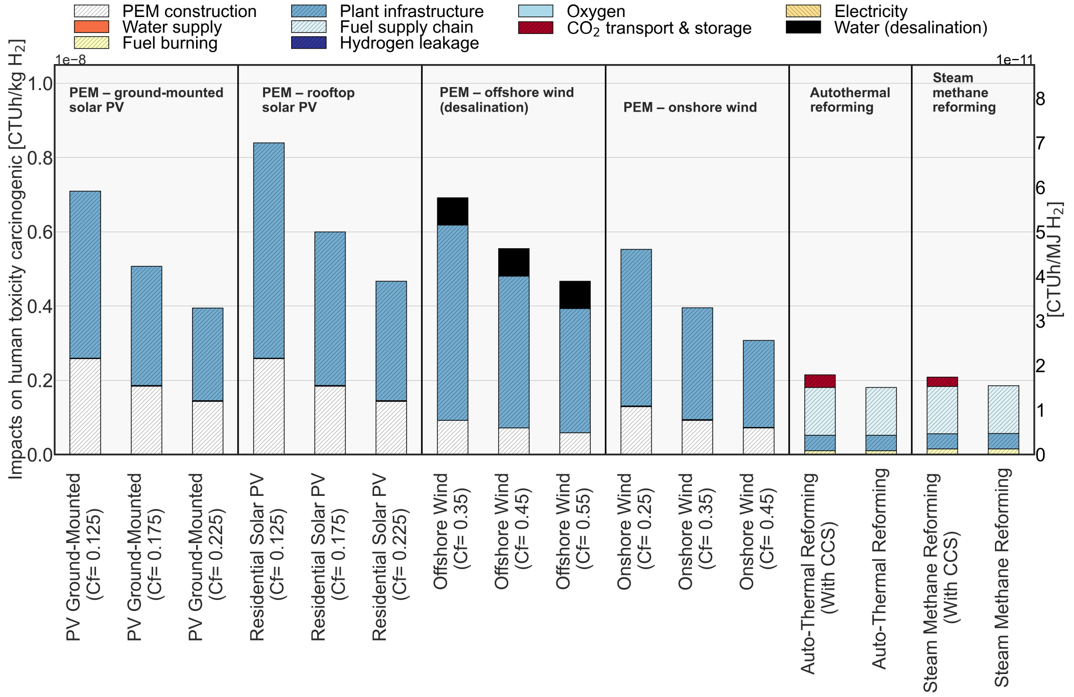

Supplementary Figure 22: Contribution analysis on human toxicity, carcinogenic. Cf = capacity factor. CCS = carbon capture and storage. PEM = polymer electrolyte membrane. PV = photovoltaic.

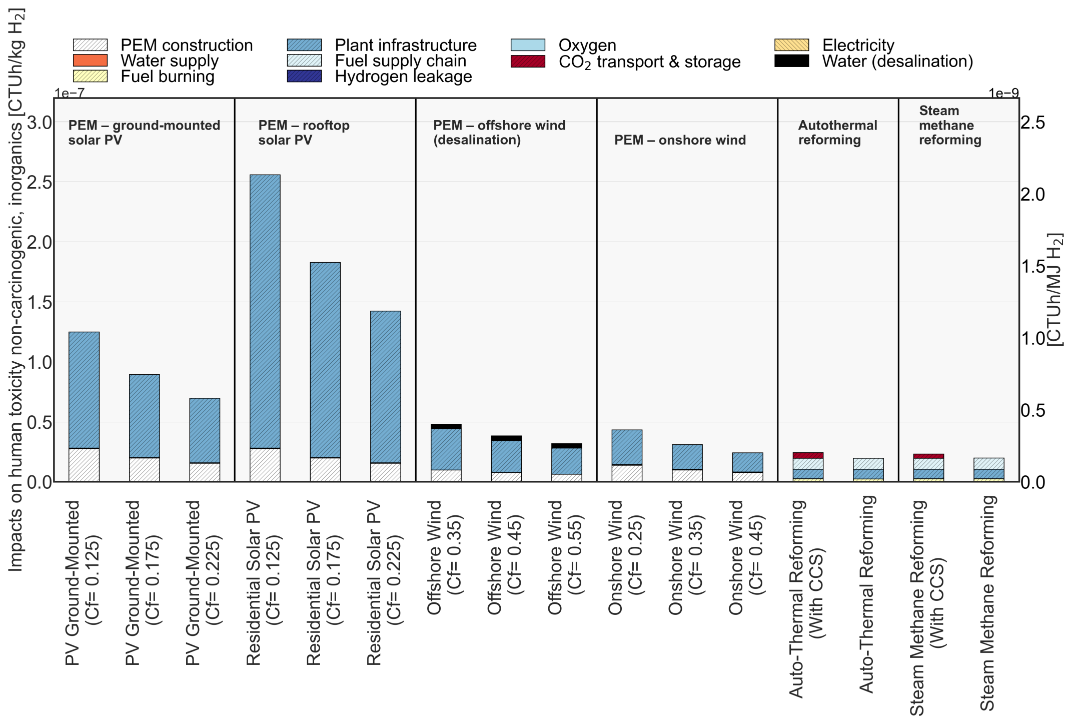

Supplementary Figure 23: Contribution analysis on human toxicity, non-carcinogenic, inorganics. Cf = capacity factor. CCS = carbon capture and storage. PEM = polymer electrolyte membrane. PV = photovoltaic.

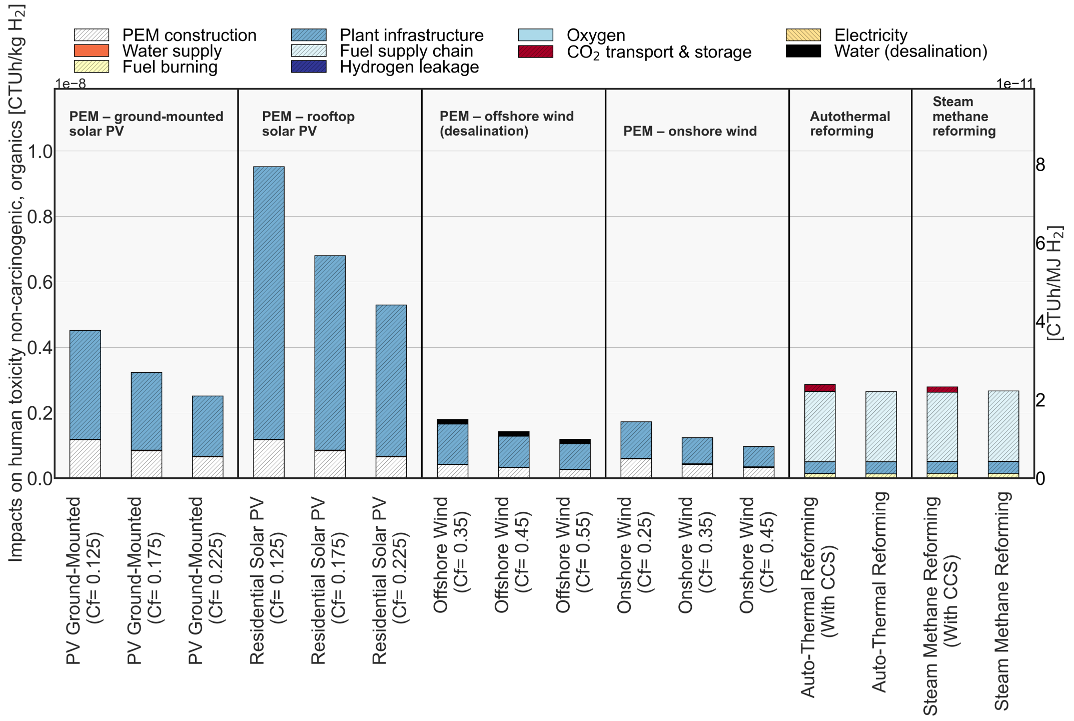

Supplementary Figure 24: Contribution analysis on human toxicity, non-carcinogenic, organics. Cf = capacity factor. CCS = carbon capture and storage. PEM = polymer electrolyte membrane. PV = photovoltaic.

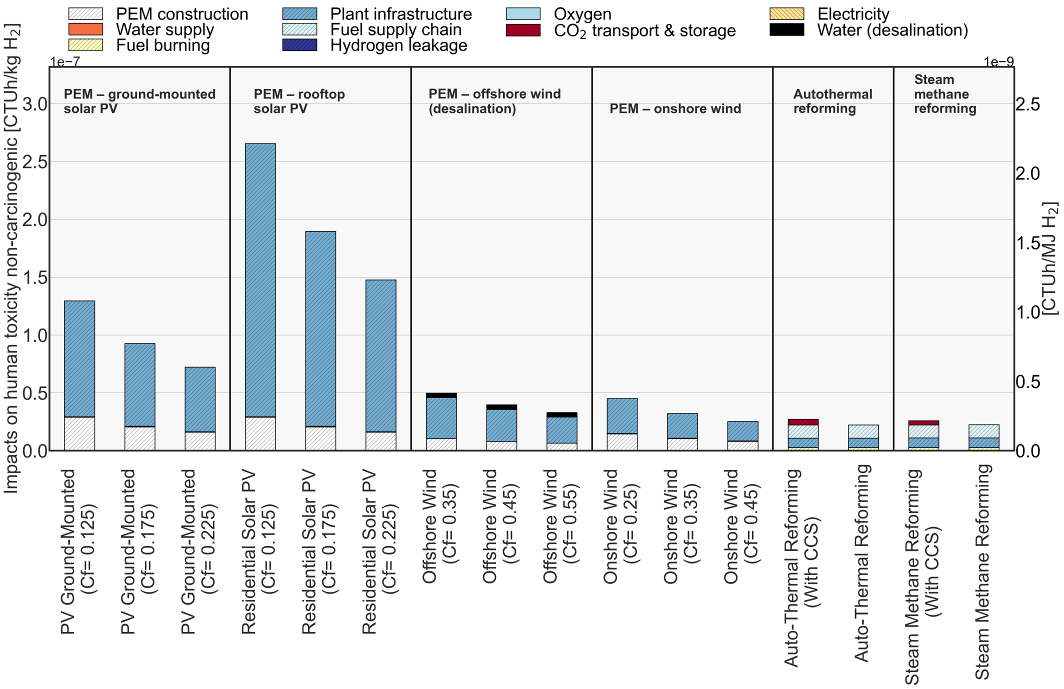

Supplementary Figure 25: Contribution analysis on human toxicity, non-carcinogenic. Cf = capacity factor. CCS = carbon capture and storage. PEM = polymer electrolyte membrane. PV = photovoltaic.

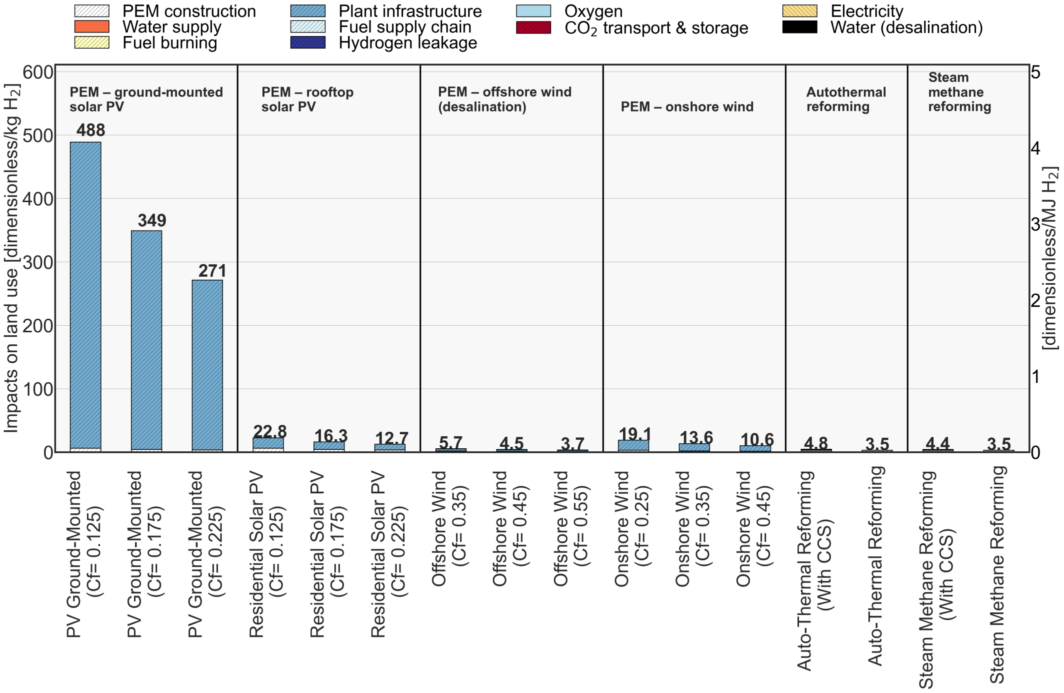

Supplementary Figure 26: Contribution analysis on land use quality. Cf = capacity factor. CCS = carbon capture and storage. PEM = polymer electrolyte membrane. PV = photovoltaic.

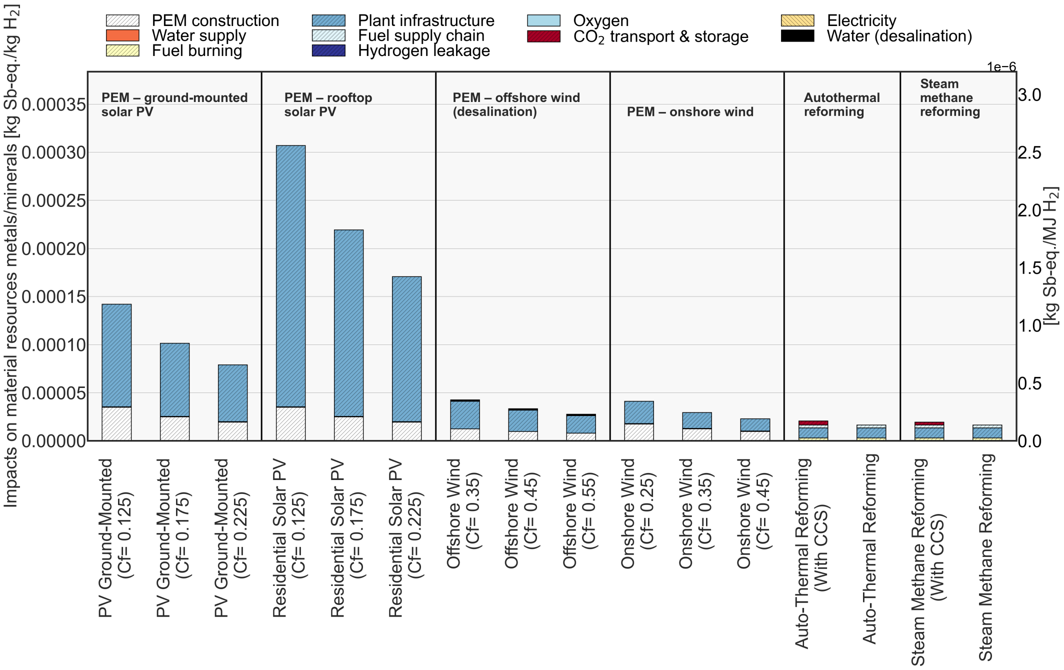

Supplementary Figure 27: Contribution analysis on metals & minerals. Cf = capacity factor. CCS = carbon capture and storage. PEM = polymer electrolyte membrane. PV = photovoltaic.

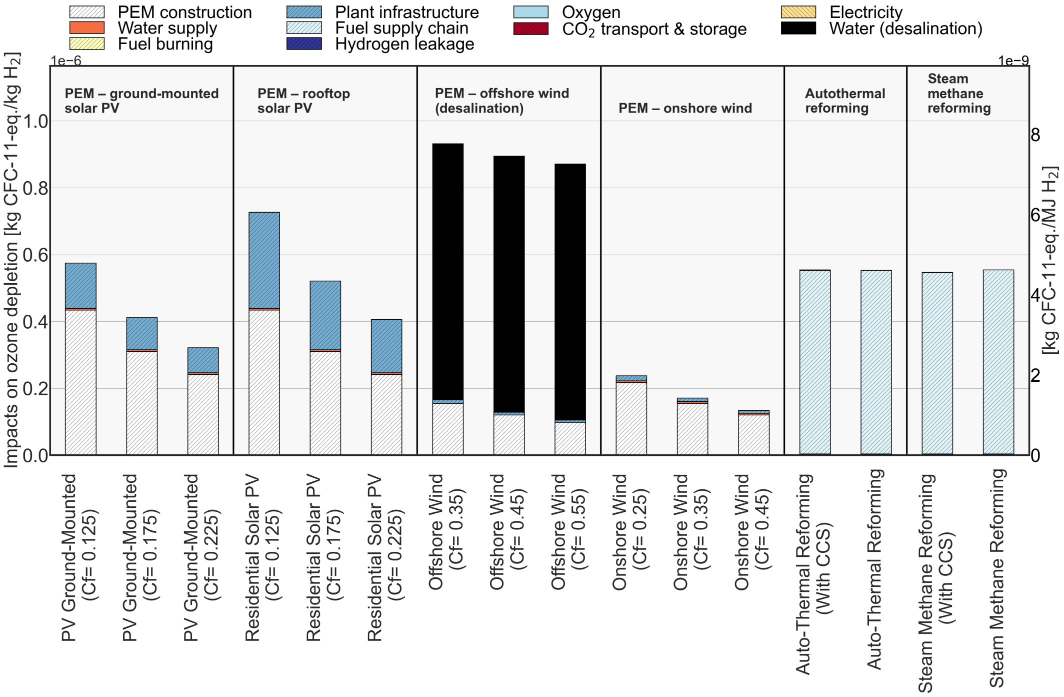

Supplementary Figure 28: Contribution analysis on ozone depletion. It is worth noting that ozone layer depletion impacts for the production of reverse osmosis membranes are tremendously high due to CFC-113 emissions, however, these impacts are likely much lower today due to the adoption of regulations such as the Montreal Protocol[46]. Cf = capacity factor. CCS = carbon capture and storage. PEM = polymer electrolyte membrane. PV = photovoltaic.

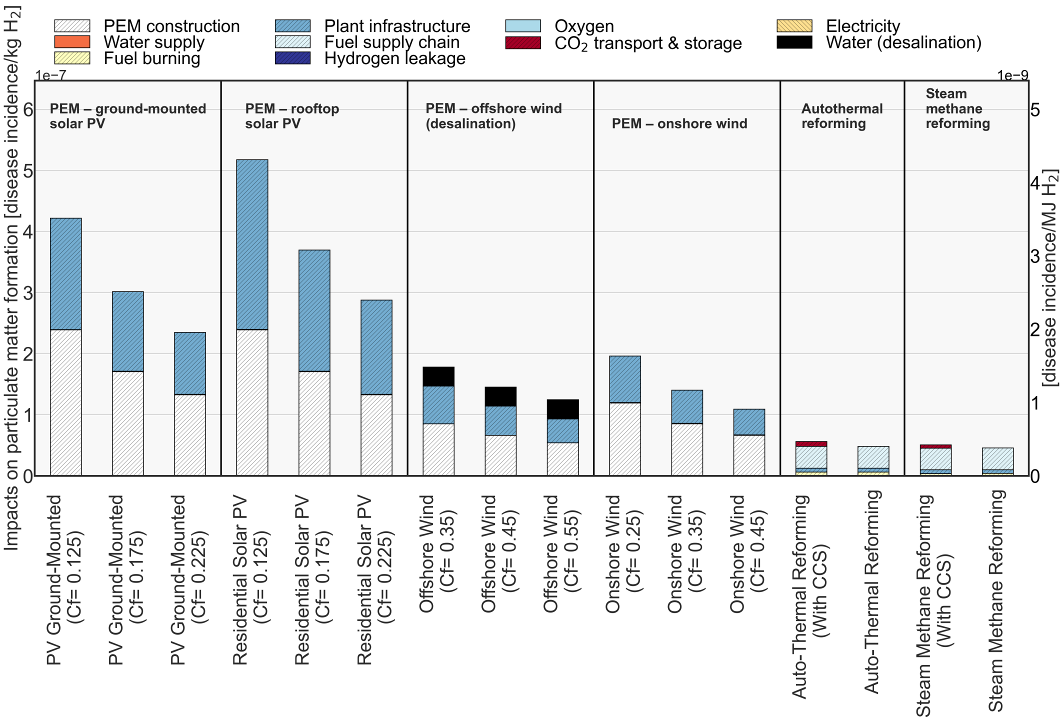

Supplementary Figure 29: Contribution analysis on particulate matter. Cf = capacity factor. CCS = carbon capture and storage. PEM = polymer electrolyte membrane. PV = photovoltaic.

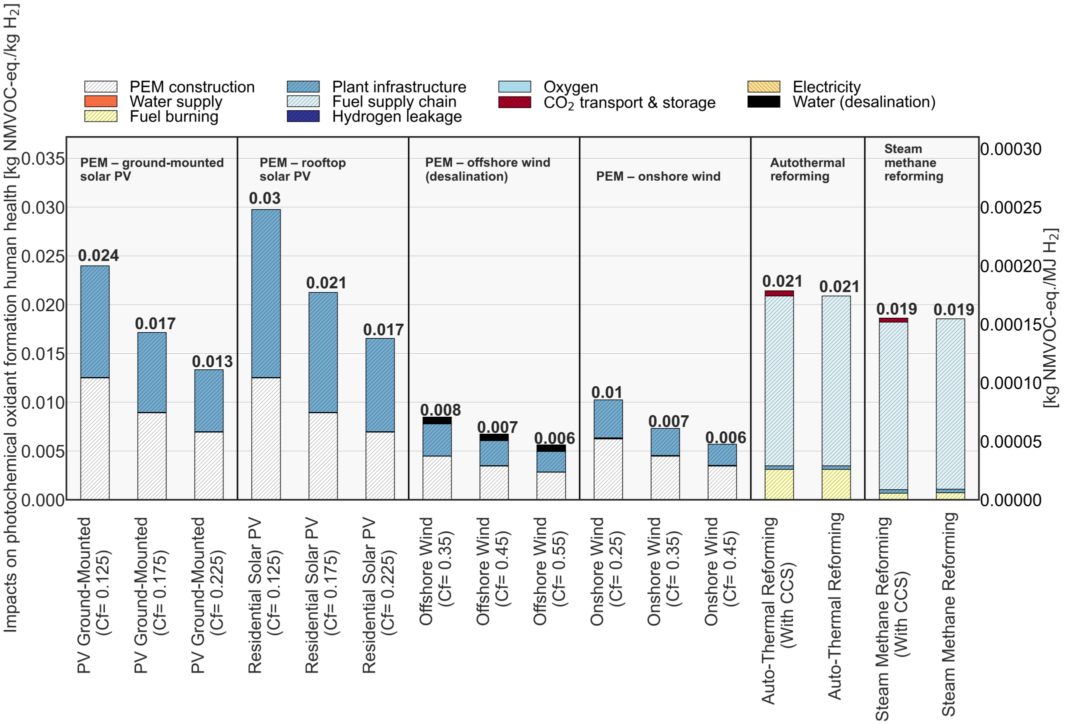

Supplementary Figure 30: Contribution analysis on photochemical oxidant formation human health. Cf = capacity factor. CCS = carbon capture and storage. PEM = polymer electrolyte membrane. PV = photovoltaic.

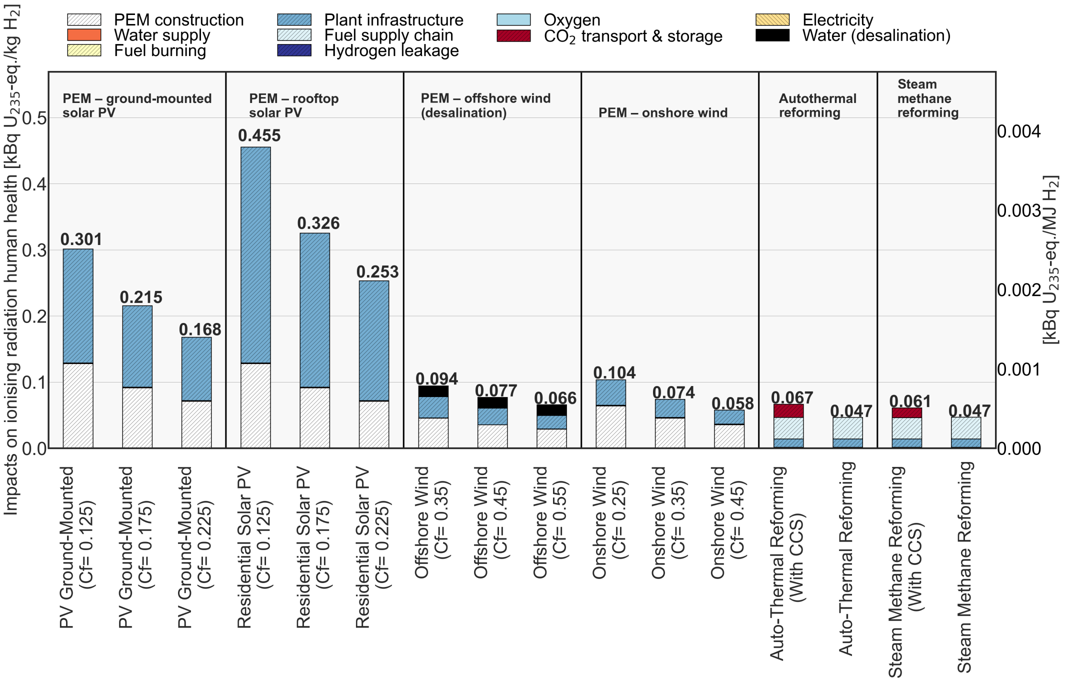

Supplementary Figure 31: Contribution analysis on ionising radiation. Cf = capacity factor. CCS = carbon capture and storage. PEM = polymer electrolyte membrane. PV = photovoltaic.

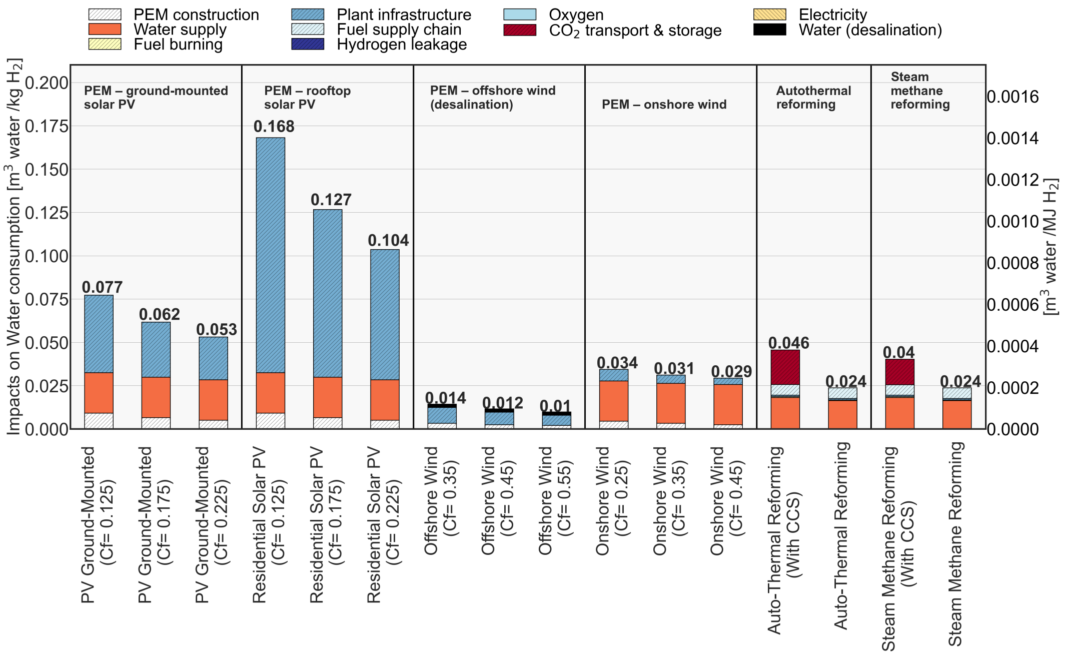

Supplementary Figure 32: Contribution analysis on water use. Cf = capacity factor. CCS = carbon capture and storage. PEM = polymer electrolyte membrane. PV = photovoltaic.

Supplementary Note 8. Additional results

Varying global land utilization factors

To consider a wider range of global land utilization factors, we provide two different sensitivity analyses using 2% and 10% of land utilization factors in Supplementary Figures 33–34. A 2% land utilization factor is considered as the lower limit and has been used as a goal in Germany for onshore wind installations[47]. The latter factor also allows the prudence of the entire hydrogen demand, in all scenarios, with electrolytic hydrogen. Further, to consider a wider range of land utilization factors, we provide two sets of land utilization factors applying 2% and 10% (similar as in Ref.[16]) of land utilization factors in Supplementary Figures 33–34. A land utilization factor of 4% has been applied in the main analysis as a scenario between low and high land utilization factors.

These figures illustrate that hydrogen production will be more concentrated with high land utilisation factors and a larger hydrogen trade is likely. In contrast, with low land utilization factors, achieving high hydrogen production quotas will be difficult, and many geographical areas must be subjected to smaller hydrogen production facilities.

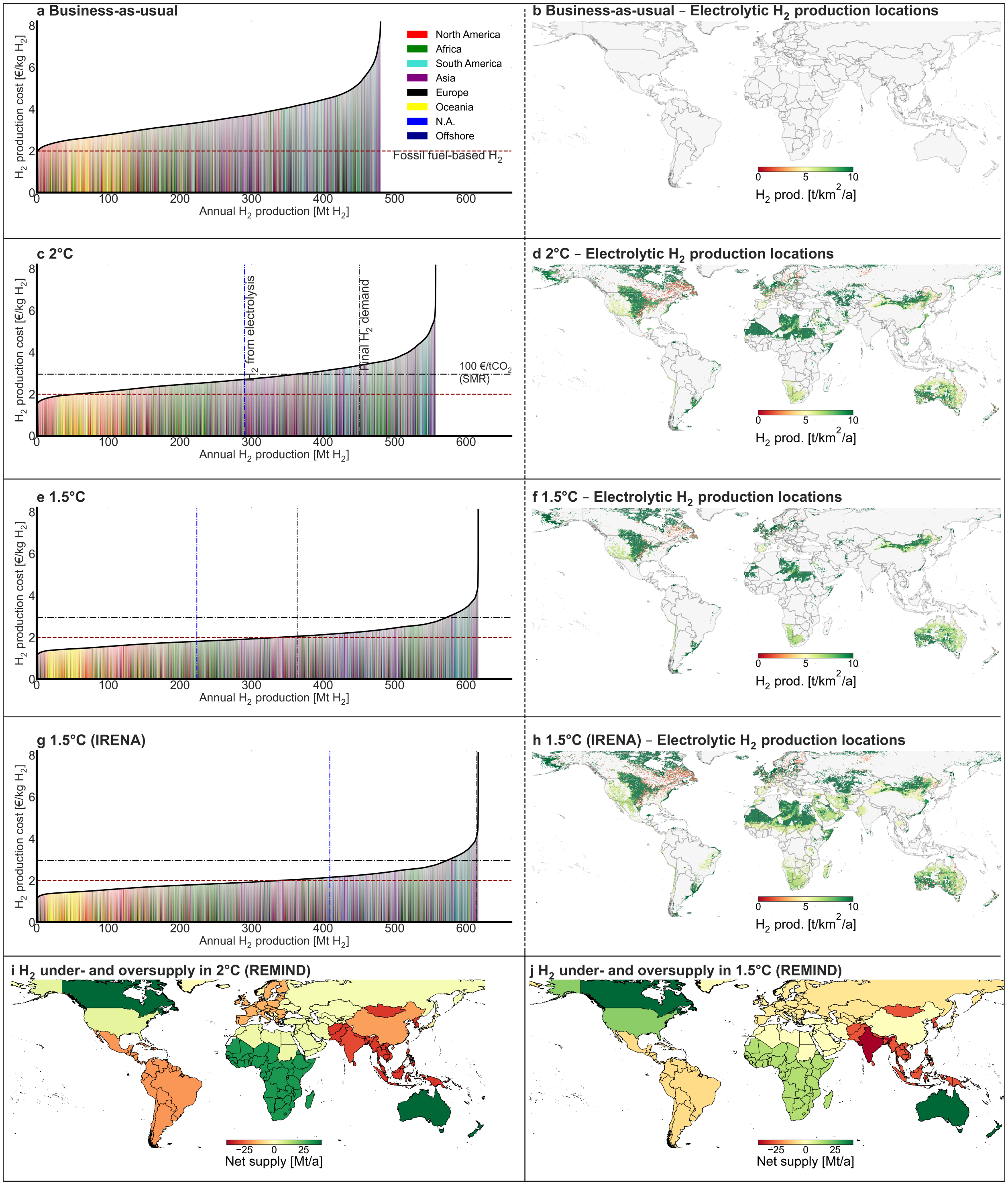

Supplementary Figure 33: Land utilization factor of max. 2%. **a,c,e,g** Cost supply curves in year 2050 for business-as-usual, 2°C, 1.5°C, and 1.5°C (IRENA), respectively. **b,d,f,h** Selected cost-optimal locations for business-as-usual, 2°C, 1.5°C, and 1.5°C (IRENA), respectively. **i–j** Net H_2 supply for 2°C and 1.5°C, respectively. N.A. = not available/applicable. IRENA = International Renewable Energy Agency. REMIND = The REgional Model of INvestments and Development.

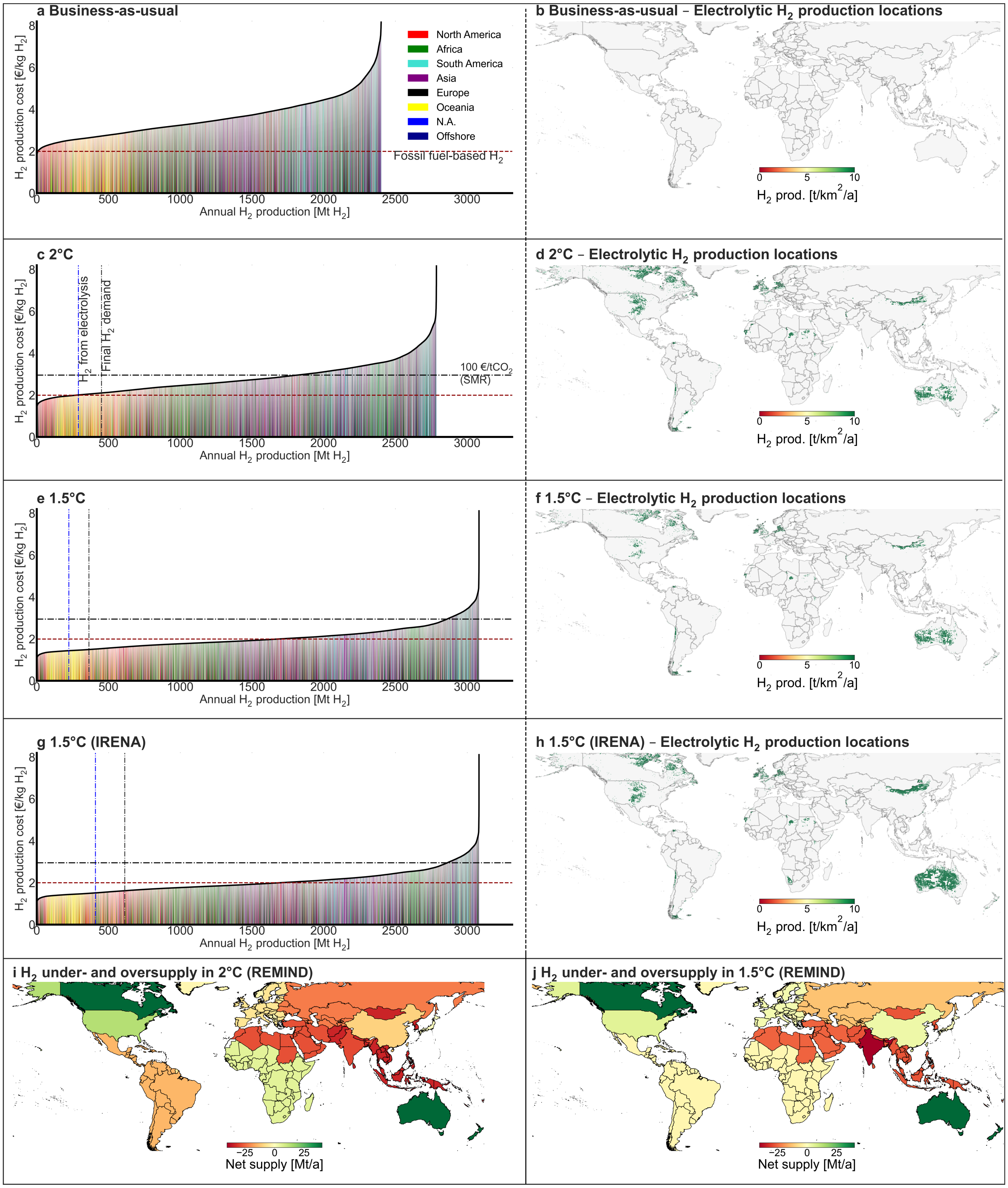

Supplementary Figure 34: Land utilization factor of max. 10%. **a,c,e,g** Cost supply curves in year 2050 for business-as-usual, 2°C, 1.5°C, and 1.5°C (IRENA), respectively. **b,d,f,h** Selected cost-optimal locations for business-as-usual, 2°C, 1.5°C, and 1.5°C (IRENA), respectively. **i–j** Net H_2 supply for 2°C and 1.5°C, respectively. N.A. = not available/applicable. IRENA = International Renewable Energy Agency. REMIND = The REgional Model of INvestments and Development.

Spider graphs of least cost case studies in selected countries

Supplementary Figure 35 shows the environmental trade-offs for different countries (on the columns) and for the different current and future scenarios considered (on the rows), normalized to the maximum impact per indicator. This figure illustrates that there are several environmental trade-offs, for example, between countries with water scarcity in combination with large solar PV energy sources (Morocco and China) and countries with abundant wind energy sources with higher critical material utilization (Canada and Denmark).

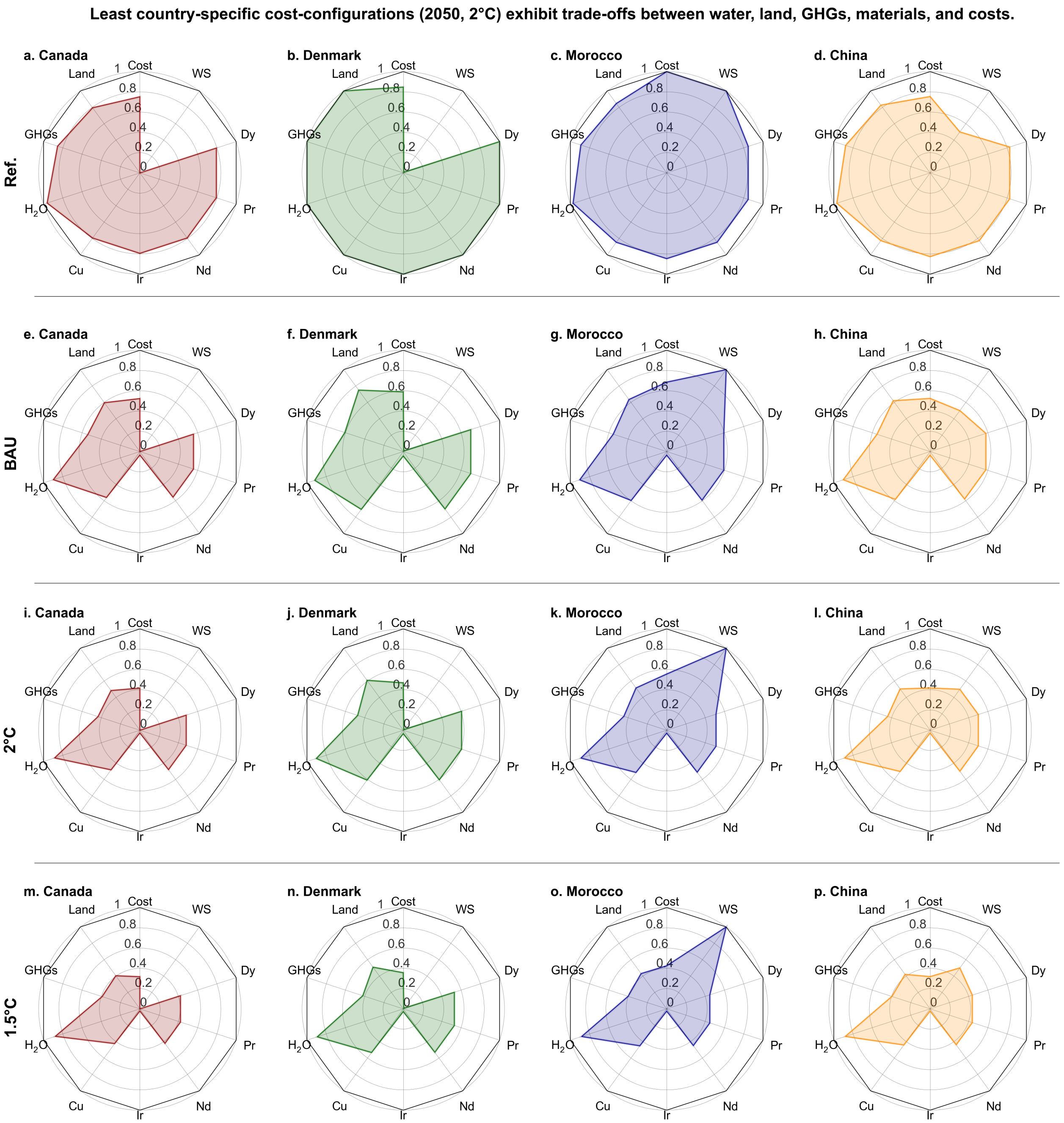

Supplementary Figure 35: Trade-offs between least cost configurations (2050, 2°C) of selected countries. **a,e,i,m** for Canada for reference, BAU, 2 °C and 1.5 °C scenarios, respectively. **b,f,j,n** for Denmark for reference, BAU, 2 °C and 1.5 °C scenarios, respectively. **c,g,k,o** for Morocco for reference, BAU, 2 °C and 1.5 °C scenarios, respectively. **d,h,l,p** for China for reference, BAU, 2 °C and 1.5 °C scenarios, respectively. WS = water scarcity. Dy = dysprosium. Pr = praseodymium. Nd = neodymium. Ir = iridium. Cu = copper. GHGs = greenhouse gases. BAU = business-as-usual. GHG = greenhouse gas.

Sensitivity analysis

Sensitivity analyses for all scenarios are presented in Supplementary Figure 36. Supplementary Figure 36**a** illustrates the influence of changing parameters by 10% (red hatched bar) and -10% (dark blue bars) on averaged global hydrogen production costs while Supplementary Figure 36**b** shows those influences on global averaged GHG emissions per kilogram of hydrogen production. It is worth noting that these figures demonstrate the influence for all global spatial locations and, thus, are "biased" or "weighted" by the distribution of these parameters across all locations. For example, if relatively more onshore wind is installed as opposed to solar PV globally, the sensitivity of onshore wind (e.g. capex) will be larger; this effect is illustrated in the reference scenario. Another point to keep in mind is that we use non-linear trend correlations resulting from an optimization problem to decide on the land area used for the installation of solar PV vs. onshore wind capacity, battery electricity storage capacity installed, and electrolyzer capacity installed. However, for this sensitivity analysis, the adjustments in parameters have not been incorporated into the initial optimization problem to establish these non-linear trends. Consequently, the outcomes of these sensitivity analyses might slightly underestimate the impact of parameter changes.

Despite these limitations, these figures clearly show the influence of parameter sensitivity on our geospatial analysis for electrolytic hydrogen production. The main article of this work discusses these results.

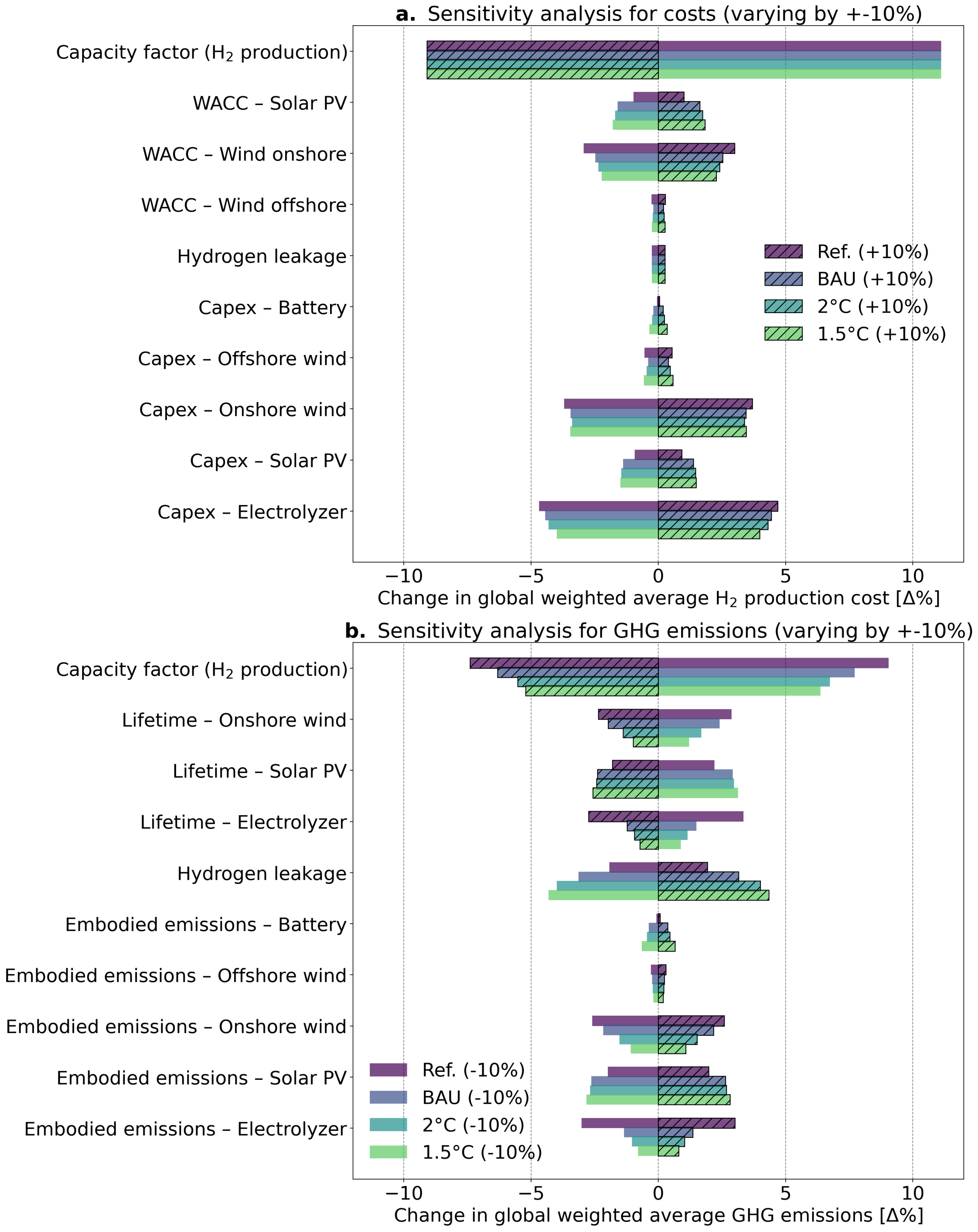

Supplementary Figure 36: Sensitivity analysis changing potentially influencing parameters by +10% and -10% for costs and GHG emissions. **a**. Sensitivity analysis to determine the influence on overall costs. **b**. Sensitivity analysis to determine the influence on overall GHG emissions. PV = photovoltaic. WACC = weighted average cost of capital. Ref. = reference case. BAU = business-as-usual. GHG = greenhouse gas.

SUPPLEMENTARY REFERENCES

1. Antonini, C. *et al.* Hydrogen production from natural gas and biomethane with carbon capture and storage - A techno-environmental analysis. *Sustainable Energy & Fuels* **4,** 2967–2986 (2020).
2. Antonini, C. *et al.* Hydrogen from wood gasification with CCS–a techno-environmental analysis of production and use as transport fuel. *Sustainable Energy & Fuels* **5,** 2602–2621 (2021).
3. Terlouw, T., Bauer, C., McKenna, R. & Mazzotti, M. Large-scale hydrogen production via water electrolysis: a techno-economic and environmental assessment. *Energy & Environmental Science* **15,** 3583–3602 (2022).
4. Terlouw, T. *et al.* Optimal economic and environmental design of multi-energy systems. *Applied Energy* **347,** 121374 (2023).
5. Terlouw, T. M. *Techno-economic and environmental assessment of low-carbon energy systems* en. Doctoral Thesis (ETH Zurich, Zurich, 2023).
6. *Agricultural land prices and rents - statistics - Statistics Explained* July 10, 2023. https://ec.europa.eu/eurostat/statistics-explained/index.php?title=Agricultural_land_prices_and_rents_-_statistics&oldid=559924#Agricultural_land_rental_prices (2023).
7. Binning, R. *Currency movements disguise global farmland performance* 2020. https://pdf.euro.savills.co.uk/uk/rural---other/spotlight-global-farmland-index---sep-2020.pdf (2023).
8. Gabrielli, P., Gazzani, M. & Mazzotti, M. Electrochemical conversion technologies for optimal design of decentralized multi-energy systems: Modeling framework and technology assessment. *Applied Energy* **221,** 557–575 (2018).
9. Holmgren, W. F., Hansen, C. W. & Mikofski, M. A. pvlib python: A python package for modeling solar energy systems. *Journal of Open Source Software* **3,** 884 (2018).
10. Holmgren, W. *et al.* *pvlib/pvlib-python: v0.9.3* version v0.9.3. Sept. 2022. https://doi.org/10.5281/zenodo.7083355.
11. Haas, S. *et al.* *wind-python/windpowerlib: Silent Improvements* 2021.
12. Terlouw, T., AlSkaif, T., Bauer, C., Mazzotti, M. & McKenna, R. Designing residential energy systems considering prospective costs and life cycle GHG emissions. *Applied Energy* **331,** 120362 (2023).
13. Gabrielli, P., Gazzani, M., Martelli, E. & Mazzotti, M. Optimal design of multi-energy systems with seasonal storage. *Applied Energy* **219,** 408 –424. ISSN: 0306-2619 (2018).
14. Huld, T., Müller, R. & Gambardella, A. A new solar radiation database for estimating PV performance in Europe and Africa. *Solar Energy* **86,** 1803–1815 (2012).

15. Weidner, T., Tulus, V. & Guillén-Gosálbez, G. Environmental sustainability assessment of large-scale hydrogen production using prospective life cycle analysis. *international journal of hydrogen energy* **48,** 8310–8327 (2023).

16. Tonelli, D. *et al.* Global land and water limits to electrolytic hydrogen production using wind and solar resources. *Nature Communications,* 14, 5532 (2023).

17. Mutel, C. L. & Hellweg, S. Regionalized life cycle assessment: computational methodology and application to inventory databases. *Environmental science & technology* **43,** 5797–5803 (2009).

18. Mutel, C. *et al.* Overview and recommendations for regionalized life cycle impact assessment. *The international journal of life cycle assessment* **24,** 856–865 (2019).

19. Kato, T., Kubota, M., Kobayashi, N. & Suzuoki, Y. Effective utilization of by-product oxygen from electrolysis hydrogen production. *Energy* **30,** 2580–2595 (2005).

20. Henriksen, M. S. *et al.* Tradeoffs in life cycle water use and greenhouse gas emissions of hydrogen production pathways. *International Journal of Hydrogen Energy* (2023).

21. Sacchi, R. *et al.* PRospective EnvironMental Impact asSEment (premise): A streamlined approach to producing databases for prospective life cycle assessment using integrated assessment models. *Renewable and Sustainable Energy Reviews* **160,** 112311 (2022).

22. Wokaun, A. & Wilhelm, E. *Transition to hydrogen: pathways toward clean transportation* (Cambridge University Press, 2011).

23. Li, J., Wei, Y.-M., Liu, L., Li, X. & Yan, R. The carbon footprint and cost of coal-based hydrogen production with and without carbon capture and storage technology in China. *Journal of Cleaner Production* **362,** 132514 (2022).

24. Gerloff, N. Comparative Life-Cycle-Assessment analysis of three major water electrolysis technologies while applying various energy scenarios for a greener hydrogen production. *Journal of Energy Storage* **43,** 102759 (2021).

25. Fayyaz, S. *et al.* Life cycle assessment of reverse osmosis for high-salinity seawater desalination process: Potable and industrial water production. *Journal of Cleaner Production* **382,** 135299 (2023).

26. Frischknecht, R. *et al. Life Cycle Inventories and Life Cycle Assessment of Photovoltaic Systems, International Energy Agency (IEA) PVPS Task 12, Report T12-19:2020* 2020.

27. ecoinvent. *ecoinvent 3.9* https://ecoinvent.org/ecoinvent-v3-9-is-released/. 2022.

28. Bareiß, K., de la Rua, C., Möckl, M. & Hamacher, T. Life cycle assessment of hydrogen from proton exchange membrane water electrolysis in future energy systems. *Applied Energy* **237,** 862–872 (2019).

29. Schmidt, T. S. *et al.* Additional Emissions and Cost from Storing Electricity in Stationary Battery Systems. *Environmental Science and Technology* **53,** 3379–3390. ISSN: 15205851 (7 2019).

30. Bauer, C *et al.* Electricity storage and hydrogen: Technologies, costs and environmental burdens. *PSI, Paul Scherrer Institut technical report* (2021).

31. (NETL), N. E. T. L. *COMPARISON OF COMMERCIAL, STATE-OF-THE-ART, FOSSIL-BASED HYDROGEN PRODUCTION TECHNOLOGIES* Nov. 19, 2023. `https://netl.doe.gov/projects/files/ComparisonofCommercialSta`[illegible] `041222.pdf` (2023).

32. *RENEWABLE POWER GENERATION COSTS IN 2022* tech. rep. (Oct. 24, 2023). `https://mc-cd8320d4-36a1-40ac-83cc-3389-cdn-endpoint.azureedge.net/-/media/Files/IRENA/Agency/Publication/2023/Aug/IRENA_Renewable_power_generation_costs_in_2022.pdf?rev=cccb713bf8294cc5bec3f870e1fa15c2` (2023).

33. *Natural gas price assumptions, 2019-2025 – Charts – Data Statistics - IEA* Oct. 24, 2023. `https://www.iea.org/data-and-statistics/charts/natural-gas-price-assumptions-2019-2025` (2023).

34. *Prices – Coal Market Update – July 2023 – Analysis - IEA* Oct. 24, 2023. `https://www.iea.org/reports/coal-market-update-july-2023/prices` (2023).

35. Agency, D. E. Technology Data—Generation of Electricity and District Heating. *Retrieved July 1st, 2023* (2023).

36. Tröndle, T., Lilliestam, J., Marelli, S. & Pfenninger, S. Trade-offs between geographic scale, cost, and infrastructure requirements for fully renewable electricity in Europe. *Joule* **4,** 1929–1948 (2020).

37. International Renewable Energy Agency, A. D. *Global hydrogen trade to meet the 1.5°C climate goal: Part I – Trade outlook for 2050 and way forward* 2022. `https://www.irena.org/-/media/Files/IRENA/Agency/Publication/2022/Jul/IRENA_Global_hydrogen_trade_part_1_2022_.pdf`.

38. Enevoldsen, P. & Jacobson, M. Z. Data investigation of installed and output power densities of onshore and offshore wind turbines worldwide. *Energy for Sustainable Development* **60,** 40–51 (2021).

39. Wiser, R. *et al.* Expert elicitation survey predicts 37% to 49% declines in wind energy costs by 2050. *Nature Energy* **6,** 555–565 (2021).

40. PIK. *Price of Hydrogen: CAPEX Data* 2023. `https://h2foroveralls.shinyapps.io/H2Dash/`.

41. International Energy Agency. *The Future of Hydrogen* tech. rep. (International Energy Agency, 2019).

42. Panos, E., Kannan, R., Hirschberg, S. & Kober, T. An assessment of energy system transformation pathways to achieve net-zero carbon dioxide emissions in Switzerland. *Communications Earth & Environment* **4,** 157 (2023).

43. Hoogwijk, M. M. *On the global and regional potential of renewable energy sources* PhD thesis (2004).

44. Dupont, E., Koppelaar, R. & Jeanmart, H. Global available wind energy with physical and energy return on investment constraints. *Applied Energy* **209,** 322–338 (2018).

45. CertifHy. *CertifHy - Developing a European Framework for the generation of guarantees of origin for green hydrogen* Accessed on Tuesday, September 31, 2021. 2016. https://www.certifhy.eu/images/media/files/CertifHy_-_definition_outcome_and_scope_LCA_analysis.pdf.

46. Hofs, B., van den Broek, W., van Eckeveld, A. & van der Wal, A. Carbon footprint of drinking water over treatment plant life span (2025–2075) is probably dominated by construction phase. *Cleaner Environmental Systems* **5,** 100079 (2022).

47. *Expanding wind energy for Germany — Federal Government* July 8, 2022. https://www.bundesregierung.de/breg-de/schwerpunkte/klimaschutz/onshore-wind-energy-act-2060954 (2024).
